# Future of snakebite risk in India: Consequence of climate change and the shifting habitats of the big four species in next five decades

**DOI:** 10.1371/journal.pntd.0013464

**Published:** 2025-09-02

**Authors:** Imon Abedin, Hey-Eun Kang, Hemanta Saikia, Won-Kyo Jung, Hyun-Woo Kim, Shantanu Kundu

**Affiliations:** 1 Dibru-Saikhowa Conservation Society, Tinsukia, India; 2 Institute of Marine Life Science, Pukyong National University, Busan, Republic of Korea; 3 Department of Basic Sciences and Humanities, College of Sericulture, Assam Agricultural University, Assam, India; 4 Research Center for Marine Integrated Bionics Technology, Pukyong National University, Busan, Republic of Korea; 5 Major of Biomedical Engineering, Division of Smart Healthcare and New-Senior Healthcare Innovation Center (BK21 Plus), Pukyong National University, Busan, Republic of Korea; 6 Department of Marine Biology, Pukyong National University, Busan, Republic of Korea; 7 Marine Integrated Biomedical Technology Center, National Key Research Institutes in Universities, Pukyong National University, Busan, Republic of Korea; 8 Ocean and Fisheries Development International Cooperation Institute, College of Fisheries Science, Pukyong National University, Busan, Republic of Korea; 9 International Graduate Program of Fisheries Science, Pukyong National University, Busan, Republic of Korea; Fundacao de Medicina Tropical Doutor Heitor Vieira Dourado, BRAZIL

## Abstract

**Background:**

Climate change is anticipated to significantly impact the biogeographic distribution of snakes, leading to notable shifts in their habitats toward anthropogenic landscapes. This may potentially increase the incidence of Big Four species (*Bungarus caeruleus*, *Daboia russelii*, *Echis carinatus*, and *Naja naja*) envenomation, a notable human-health risk that has not yet been assessed in India being the most affected country in South Asia. Therefore, this study integrates species distributions with socioeconomic and healthcare data to prioritize areas for targeted interventions to mitigate the envenomation risks effectively in India.

**Methodology/Principal findings:**

The present study employed ensemble species distribution models (SDMs) to analyze the geographical distribution of the Big Four species under current climatic conditions and projected these models to estimate potential species distributions up to 2080. Furthermore, by incorporating various future climatic scenarios, the study assessed the potential loss and gain of suitable habitats along with their overlap with cropland and built-up areas. Further, integrating SDMs with socioeconomic scenarios and present health infrastructure, the study developed a risk index to estimate the current and upcoming vulnerable districts and states in next five decades. The results indicate significant losses in potentially suitable habitats for the Big Four species under future climatic scenarios. However, the risk index identified several southern Indian states and districts, such as Karnataka (Chikkaballapura, Haveri, and Chitradurga etc.) and Gujarat (Devbhumi Dwarka and Jamnagar etc.), as having high vulnerability to snakebite. Additionally, under climate change scenarios, many northern and northeastern states and districts, including Assam (Nagaon, Morigaon, and Golaghat etc.), Manipur (Tengnoupal), and Rajasthan (Pratapgarh), have experienced an increased risk of snakebite, presenting a significant public health concern in these regions.

**Conclusion/Significance:**

The integrated risk index indicates that the southwestern region urgently needs priority attention to combat fatalities from envenomation by the Big Four species, while also highlighting the future needs of the northern and northeastern region to aid public health efforts. To mitigate these impacts, local governments and international communities must intensify efforts to counter climate change and protect vulnerable regions from Big Four envenomation.

## Author summary

Snake envenomation is a critical public health issue in India, disproportionately affecting economically deprived populations. India records the highest number of snakebite fatalities worldwide, primarily due to the Big Four species. While previous research has focused on reducing snakebite-related deaths, little has been done to integrate species distribution with socioeconomic factors, anthropogenic landscape, and healthcare infrastructure to assess district-level risks in India. This study is the first to predict the impact of climate change on the distribution of the Big Four species while identifying regions most at risk. The research incorporates socioeconomic data and healthcare infrastructure to prioritize regions for medical intervention now and in the future. Addressing this emerging threat will require coordinated efforts from government authorities, regional health organizations, and the international community to effectively manage snake envenomation as a neglected tropical disease (NTD) in India.

## Introduction

In recent times, human activities such as habitat destruction have significantly increased human-wildlife interactions and raised the risk of fatalities and disease transmission through vectors species [1–4]. The Neglected Tropical Diseases (NTDs), caused by venomous snakes from the Viperidae and Elapidae families poses significant threat to humans and lead to more fatalities than any other vertebrates [3,5]. Despite many significant strategies adopted by local and global authorities, snakebite has still not received adequate attention through integrated approaches, thus justifying its classification as a NTD [6]. Snakebite envenoming (the injection of venom into the body) is a significant public health challenge due to its high mortality and morbidity rates, primarily caused by acute injuries and long-term complications [7–12]. Furthermore, accurate incidence data for snakebites, particularly in rural tropical regions where they are most prevalent, are scarce. The reliable data are predominantly available from a few developed countries where snakebites are less common. Therefore, the true global incidence, impact, and characteristics of snakebite envenoming across different regions remain largely unknown [6]. This gap prompted the World Health Organization (WHO) to emphasize the urgent need for stronger and more comprehensive evidence on the health and socioeconomic impacts of snakebites in developing and underdeveloped countries through its 2021–2030 NTD Roadmap [13,14]. The WHO estimates that annually, between 4.5 and 5.4 million snakebites occur worldwide, resulting in 81,000–138,000 fatalities, with approximately three times that number surviving but often experiencing amputations and permanent disabilities [15,16]. Furthermore, many deaths and severe outcomes resulting from snakebite envenomation are preventable through timely access to safe and effective antivenoms [7,17]. Hence, in 2017, the WHO included snakebite envenoming in its priority list of NTDs. In 2019, the WHO initiated a comprehensive strategy for the prevention and control of snakebites, targeting a 50% reduction in deaths and severe disabilities by 2030 compared to the 2015 baseline [15]. This strategy emphasizes the establishment of a revolving stockpile of antivenoms for rapid deployment to high-risk areas and assisting countries in designing and implementing locally relevant plans that align with national health policies [18].

Specifically, snakebite-induced deaths and disabilities is very prevalent in Asia, largely due to limited healthcare infrastructure and a lack of effective antivenoms. This region has the highest global incidence, with over 1 million bites annually, leading to around 45,000–50,000 deaths and morbidity rates nearly four times higher than mortality [8,15,19]. However, discrepancies in epidemiological data on snakebite envenomation and deaths in Southern Asian countries arise from a lack of coordinated efforts and systematic analyses to accurately determine the number of snakebites in this region [20]. Consequently, India is particularly vulnerable, accounting for approximately half of the global snakebite deaths [8,15]. The direct estimation of 46,000 annual snakebite deaths in India in 2005 prompted a revision of the WHO’s global estimate, which had previously suggested a similar number for the entire world [21]. The 2005 estimate for India was based on an analysis of approximately 123,000 verbal autopsy records from 2001 to 2003, part of the Registrar General of India’s Million Death Study (MDS), one of the largest nationally representative mortality surveys. Currently, the MDS has reported cause-specific mortality patterns for over 600,000 deaths across India from 2001 to 2014 [15,22]. Additionally, high snake densities are often found in grain-producing areas, where they are drawn by large prey populations of rodents and amphibians. These ecological dynamic increases snakebite incidents in fields, storage facilities/nearby buildings [15,23]. These incidents in the Indian scenario are primarily attributed to the Big Four species (Indian Common Krait *Bungarus caeruleus*, Indian Russell’s Viper *Daboia russelii*, Indian Saw-scaled Viper *Echis carinatus*, and Indian Spectacled Cobra *Naja naja*), which are responsible for over 90% of all envenomations in the country. The vulnerability assessments by previous studies indicate exposure to these snakes, which lack effective antivenom treatments [8,24,25]. The Big Four species are thus considered the most medically significant and dangerous snake species in the region, believed to cause the vast majority of snakebite fatalities. Moreover, while other venomous species are known to cause lethal envenoming, their bites are relatively rare and lead to a much smaller number of deaths each year compared to the Big Four species [26,27].

Furthermore, climate change is impacting global biodiversity by altering species geographic ranges, with region- and species-specific shifts resulting in expansions, contractions, or shifting ranges [14,28,29]. Such changes may increase human-snake interactions across rural and urban areas, presenting new challenges for public health and medical management [7,13,14,30–33]. Consequentially, to mitigate snakebite risk in affected regions, it is essential to implement strategies that enhance decision-making in healthcare delivery, antivenom research, and production capabilities. Additionally, these strategies should address current and anticipated hotspots of human-snake interactions, considering future environmental changes [33]. Therefore, addressing this challenge has seen the effective adoption of species distribution modeling (SDM) on a global scale to assess the dynamics of species distributions due to climate change [14,32,34,35]. Numerous studies have employed modeling approaches to analyze the geographical ranges of venomous snakes and predict how these ranges may shift in response to future climatic and geographical conditions [14,36,37]. Given the potential for climate change to affect the distribution of venomous snakes, researchers frequently utilize predictive modeling techniques to anticipate changes in the geographical ranges of species that pose significant public health risks [14]. Nevertheless, most of these studies have focused on specific countries in Asia (such as Bangladesh, Nepal, Iran etc.) and other continents around the world [14,32,34,35,38,39].

Specifically, India is the most vulnerable country in Asia and responsible for nearly half of the global snakebite fatalities, lacks comprehensive research on assessing the geographic shifts of the Big Four species, which raises concerns among both rural and urban populations. Therefore, to address this deficiency, the current study employs integrated SDM approach with the following objectives: (i) to assess the present distribution of the Big Four species and their future dynamics under various climate change scenarios in India; (ii) to evaluate the shifts in agricultural and built-up areas that overlap with their suitable habitats in both present and future; and (iii) to develop a risk index for each district and state for each of the Big Four species in present and future scenarios. Hence, integrating the distributional shifts of the Big Four species with their overlap in agricultural fields and built-up areas in both rural and urban settings, along with developing a risk index that includes socioeconomic factors and health infrastructure, will help prioritize specific areas for government snakebite-related policy interventions. This approach will provide a comprehensive overview of risk zones for the Big Four species in both current and future environmental conditions. Therefore, addressing these targeted contexts can effectively reduce the mortality and morbidity associated with snake envenomation in India.

## Materials and methods

### Ethics statement

The present study relied entirely on secondary data sources from the International Union for Conservation of Nature (IUCN) Geospatial Conservation Assessment Tool (GeoCAT) website (https://geocat.iucnredlist.org/) and GIS-based analyses. No field sampling was conducted, and no animals were captured or handled at any stage of the research. As a result, ethical approval from an institutional ethics committee was not required.

### Study area and data collection

The Big Four venomous snake species share similar IUCN ranges across the Asian continent, predominantly overlapping in South Asian countries such as India, Nepal, Sri Lanka, Bangladesh, and Pakistan [40–43] (S1 Fig). However, *E. carinatus* has a broader distribution extending into the Middle East, including Saudi Arabia, Iran, and Turkmenistan (S1 Fig). The majority of the Big Four species distribution range converges in India, making it especially vulnerable and contributes significant numbers of snakebite fatalities [8,15,40]. Consequently, India has been selected as the primary focus of this study due to the significant overlap of all four species within its mainland borders. Accordingly, occurrence records for the Big Four species in the study were compiled using the IUCN Geospatial Conservation Assessment Tool (GeoCAT), which aggregates data from two major open-access biodiversity repositories, viz., GBIF and iNaturalist along with human observations and literature sources [44]. These platforms provide extensive species occurrence data covering a wide range of taxa, geographic regions, and temporal scales. Their credibility is reinforced through detailed metadata, expert validation, community-based vetting, and alignment with curated datasets. Hence, integrating data from both sources, this approach yields a comprehensive and spatially robust dataset, well-suited for species distribution modeling and conservation planning. The locations gathered from these sources span the temporal range from post-2005 to the present, relying solely on direct sightings and evidence while deliberately excluding captive records or museum specimens. The model ultimately included 388 location points for *B. caeruleus*, 592 for *D. russelii*, 337 for *E. carinatus*, and 667 points for *N. naja*, respectively (S1 Fig) [45]. Additionally, these location points were spatially rarefied within 1 km^2^ using the spatial rarefication function in SDM Toolbox v2.4 which is adequate for performing SDMs [46].

### Selection of covariates

A comprehensive set of species-specific covariates, encompassing bioclimatic, topographic, habitat, and anthropogenic variables, was selected to analyze their effects on the on the habitat suitability of the big four species in the study area (S1 Table). These variables were selected to capture the complex drivers of species distributions, which are shaped by climatic conditions, topographic features, habitat characteristics, and human impacts. Thus, incorporating this diverse set of predictors improves the ecological realism and predictive performance of distribution models [47]. The bioclimatic variables (n = 19) which determines the environmental conditions were sourced from the WorldClim website (https://www.worldclim.org/) [35]. Meanwhile, the topographic variables (elevation, slope, and aspect) were obtained from the SRTM website (http://srtm.csi.cgiar.org/srtmdata/) at a spatial resolution of 90 meters. Furthermore, the habitat variables for the Big Four species were chosen based on their habitat type classification as defined by the IUCN Red List assessment criteria [40–43]. The ESRI Sentinel-2 10-Metre Land Use/Land Cover (LULC) 2023 raster was downloaded from the ESRI Living Atlas website (https://livingatlas.arcgis.com/landcover/). The dataset was available in tiles that was merged to cover the mainland India, ensuring comprehensive spatial coverage. The LULC data contained several land cover classes and was in categorical format. However, to extract each habitat layer from the original LULC dataset, the relevant habitat categories were individually reclassified using the Reclassify tool in ArcGIS v10.6. In this process, only the target habitat category was retained, while all other categories were removed by assigning them ‘NoData’. This approach allowed each habitat type to be isolated into a distinct layer, ensuring that the targeted habitat was clearly separated for further analysis. The resultant habitat layers isolated into individual categories were tree cover (abbreviated as euc_124) and rangeland/barelands (abbreviated as euc_20) which represent the habitats of the Big Four species as outlined in the recent IUCN assessments. Additionally, anthropogenic variables viz. cropland (abbreviated as euc_40) and built-up (abbreviated as euc_built) were also extracted from the same ESRI LULC data and reclassified accordingly (S1 Table). Given that these variables were initially in categorical form, they were converted into continuous variables through the Euclidean Distance function in ArcGIS v10.6. The Euclidean Distance function for a raster calculates the straight-line distance from each cell to the nearest feature or specified point, creating a continuous surface that represents spatial proximity. This method is used to assess the relative distance of each raster cell to environmental variables or species occurrences, aiding in habitat suitability modeling. Subsequently, all variables were resampled to a spatial resolution of 2.5 minutes (~4.5 km²) using the Spatial Analyst extension in ArcGIS v10.6 to balance spatial precision with computational feasibility, ensuring manageable processing across the extensive study area (S1 Table) [48]. The spatial multicollinearity among the variables was rigorously tested using the SAHM (Software for Assisted Habitat Modelling) package integrated within the VisTrails software [49]. The variables with a Pearson correlation coefficient (r) exceeding 0.8 were excluded from further analysis to minimize redundancy and enhance model reliability. These same variables were also evaluated using Spearman and Kendall correlation coefficients to further reduce the likelihood of inter-variable correlation. Consequently, for each highly correlated pair within a variable category, one variable was retained based on its ecological relevance, broader temporal representation, and high contribution. This systematic approach ensured that only uncorrelated and ecologically meaningful predictors were included in the final modeling process [50] (S2–S5 Figs). Furthermore, a qualitative assessment of suitable habitats across various states within the species distribution ranges was meticulously conducted using the zonal statistics function in ArcGIS v.10.6 [48,51].

### Species distribution modelling

**Fig 1:**
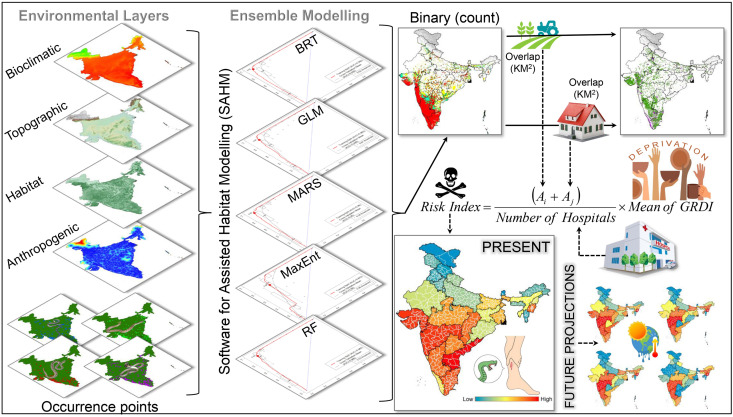
This figure provides a comprehensive flow diagram outlining the methodology of the study, including each major step and process leading to the derivation of the risk index. BRT: boosted regression tree; GLM: generalized linear model; MARS: multivariate adaptive regression spines; MaxEnt: Maximum entropy; RF: Random Forest; GRDI: Global Gridded Relative Deprivation Index; *A*_i_: overlapping area of cropland (in sq. km.); *A*_j_: overlapping area of built-up (in sq. km.). The administrative layer of the map was obtained from the DIVA-GIS website (https://diva-gis.org/data.html). The base layer of the maps is an elevation raster sourced from the SRTM website (http://srtm.csi.cgiar.org/srtmdata/) and was created using ArcGIS software. The species photographs were provided by Anirban Chaudhuri and Raju Vyas through personal communication and were used with their prior permission.

The distribution models were meticulously evaluated using an ensemble approach that incorporated multiple modeling algorithms to develop the final distribution models for each species. Specifically, five distinct algorithms were employed: Maximum Entropy (MaxEnt), Random Forest (RF), Boosted Regression Tree (BRT), Generalized Linear Model (GLM), and Multivariate Adaptive Regression Splines (MARS) [47,52,53]. The selection of these models captures various aspects of species-environment relationships. Specifically, MaxEnt handles presence-only data, BRT and RF manage non-linear interactions with high accuracy, while GLM and MARS offer interpretability and flexibility, creating a comprehensive modeling approach [54]. These algorithms were implemented within the VisTrails software utilizing the SAHM package [49,55]. The model evaluation criteria included an Area Under the Curve (AUC) threshold of 0.75 [56–58]. Hence, the software generated two output layers, i.e., a probability surface suitability layer ranging from 0 (low) to 1 (high) and an ensemble count map ranging from 0 to 5, where each pixel represented number of model agreement on suitability. The Sensitivity = Specificity (SES) threshold was applied to convert continuous habitat suitability outputs into categorical presence-absence predictions. The category/class “5” represents pixels where all five models were in agreement, thereby indicating suitable habitat extent. Hence, to determine the total area in sq. km., the number of pixels classified as “5” was multiplied by the raster cell size/spatial resolution. Additionally, various evaluation metrics, such as Area Under the Curve (AUC and ΔAUC), True Skill Statistic (TSS), Cohen’s Kappa, Proportion Correctly Classified (PCC), specificity, and sensitivity, were calculated to thoroughly assess and compare model performance using both the training data and cross-validation sets (n = 10) [59–62]. The future projections were conducted under potential climate change scenarios using two distinct shared socio-economic pathways (SSPs): SSP245 and SSP585, covering the periods 2041–2060 and 2061–2080. The SSPs are scenarios employed in climate change research to investigate future socioeconomic conditions and their effects on greenhouse gas emissions and climate impacts. The SSP245 illustrates a future with moderate mitigation and adaptation efforts. In contrast, SSP585 envisions a future characterized by high greenhouse gas emissions and minimal adaptation efforts, assuming rapid population growth, high energy demand, and minimal environmental regulation, resulting in persistent emission increases throughout the century. The selection of SSP245 and SSP585 ensures a comprehensive assessment by representing both moderate and high-emission trajectories, thereby capturing a wider spectrum of potential climate outcomes [14]. This approach provides a more robust framework for evaluating future risks compared to other scenarios, which may either overly underestimate or overestimate emissions, thus offering a more realistic projection of potential climate impacts. The Hadley Centre Global Environment Model in Global Coupled Configuration 3.1 (HadGEM3-GC31 LL) from the sixth Coupled Model Intercomparison Project (CMIP6) was selected for these projections due to its acknowledged performance in South and Southeast Asia [51,63–65]. This model is among the high-performing CMIP6 simulations for the region, offering improved representation of large-scale climatic patterns [66,67]. Additionally, the non-climatic raster data, which include various habitat types and anthropogenic elements (such as tree cover, rangelands/barelands, cropland, and built-up areas), were kept constant in future projections. This approach was taken because accurately projecting their growth is challenging, however it helps to isolate the impact of climate change [48] (Fig 1).

### Data preparation for risk index

Since most snakebite incidents in anthropogenic landscapes occur within cropland and built-up areas, the first step involved calculating the overlap (in square kilometers) between the suitable areas of the Big Four snake species and these land-use types. This was achieved using the Raster Calculator tool in ArcGIS v10.6 [15,23,68]. To account for socioeconomic vulnerability, the Global Gridded Relative Deprivation Index (GRDI) v1 (2010–2020) was utilized, that was obtained from NASA’s Socioeconomic Data and Applications Center (SEDAC) (S6 Fig) [69]. The GRDI integrates multiple indicators, including population density, educational attainment, healthcare access, infrastructure quality (water, sanitation, electricity), housing conditions, and digital connectivity, providing a comprehensive measure of deprivation relevant to snakebite risk [70,71]. The healthcare facility data for India were sourced from the United Nations Office for the Coordination of Humanitarian Affairs (OCHA), which aggregates health infrastructure information from OpenStreetMap (OSM) to support humanitarian aid, disaster response, and public health planning [8].

## Risk index formulation

The formulation of the Risk Index for Big Four species involves three key components: (i) the overlapping suitable areas with cropland and built-up regions, (ii) the number of healthcare facilities, and (iii) the mean GRDI of the region. The Risk Index is derived by first summing the overlapping areas of cropland and built-up land, as a greater overlap indicates an increased likelihood of high snakebite incidents. Moreover, the socioeconomic vulnerability of a region as reflected by the GRDI can further exacerbate the risk, as areas with limited basic facilities may struggle to manage snakebite cases effectively. Therefore, the summed overlap is multiplied by the mean GRDI to account for these socioeconomic factors. Finally, the availability of healthcare facilities plays a crucial role in mitigating the consequences of snakebites. The regions with a higher number of healthcare facilities are better equipped to manage snakebite-related emergencies, thereby reducing associated mortality and morbidity. As a result, the combined risk is normalized by dividing by the number of healthcare facilities.

The final formula is:

$Risk\;Index=\frac{\left(A_{i}+A_{j}\right)}{Number\;of\;Hospitals}\times Mean\;of\;GRDI$

where *A*_*i*_ is the overlapping area of cropland (in sq. km.) and *A*_*j*_ is the overlapping area of built-up (in sq. km.).

Moreover, this Risk Index was calculated for each district and state across mainland India under both present and future projected climate scenarios. An equal-weighted model was adopted to construct the Risk Index for snakebite risk across four targeted species. The equal-weighting approaches are commonly recommended in environmental risk assessment and composite index development, particularly when empirical evidence does not support differential weighting among components [72,73]. In this study, cropland and built-up area overlaps were treated as equally significant drivers of human-snake interactions, given that both land-use types can facilitate snakebite risk. Thus, assigning equal weights to these factors avoids arbitrary bias and ensures that each type of exposure is considered with equal importance, thereby enhancing the comparability and reproducibility of the model across geographic and ecological contexts [74]. Likewise, GRDI (a composite index for socioeconomic vulnerability) and healthcare infrastructure (measured as the number of hospitals) were incorporated without additional weighting, in recognition of their complementary roles in shaping risk. Hence, the risk index was developed using an equal-weighting approach by integrating the overlapping areas of cropland, built-up regions, socioeconomic conditions, and the number of healthcare facilities. This choice was made to maintain methodological transparency and consistency across all components, especially in the absence of robust empirical justification for assigning differential weights to these diverse factors contributing to snakebite risk. The equal-weighted models are further favored in multidimensional assessments for their transparency, interpretability, and methodological simplicity [75]. Additionally, this approach provides a flexible foundation that can be refined in future iterations as more localized or species-specific data become available. Furthermore, a sensitivity analysis was also performed for the Risk Index of each of the Big Four snake species in district and state level to assess their vulnerability to changing climatic conditions.

## Results

### Ensemble model performance

**Fig 2:**
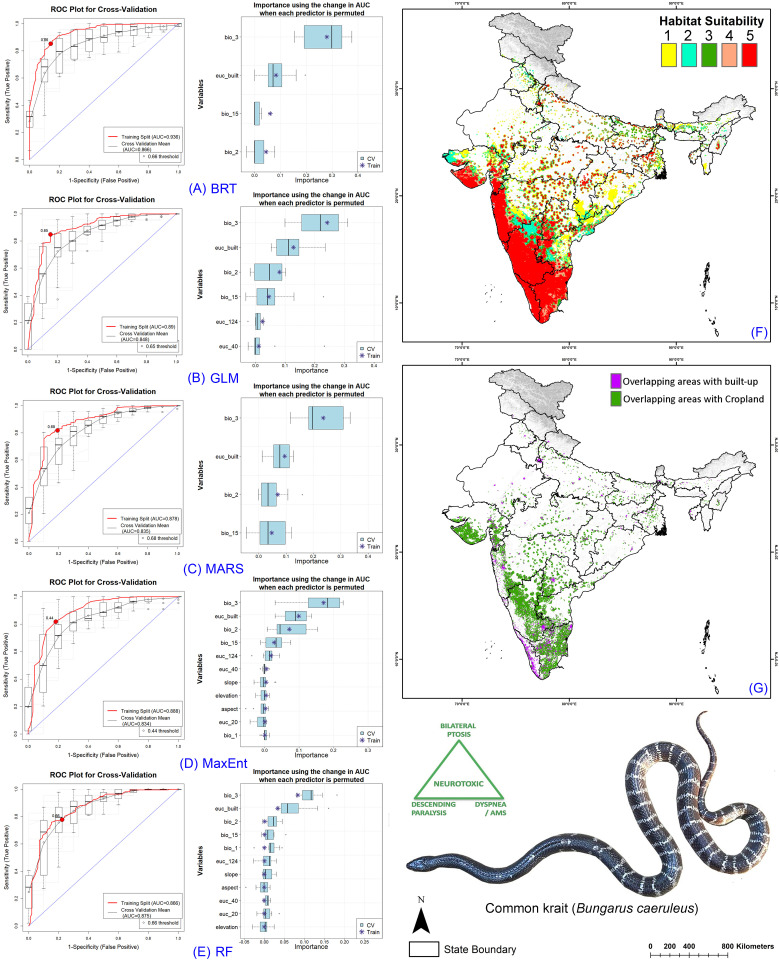
Model evaluation plot, showing the average training ROC of both training and cross-validation (CV) and variables selected by the models for the replicate runs under five models of *B. caeruleus.* (A) showing ROC plot of boosted regression tree (BRT), (B) generalized linear model (GLM), (C) multivariate adaptive regression spines (MARS), **(D)** Maximum entropy (MaxEnt), **(E)** Random Forest (RF), **(F)** The suitable habitat extent in the present scenario where the ‘class 5’ determines the extremely suitable habitat extent, and **(G)** The overlapping cropland and built-up/urban areas with the suitable extent (Class 4 and Class 5) in the present scenario. The administrative layer of the map was obtained from the DIVA-GIS website (https://diva-gis.org/data.html). The base layer of the maps is an elevation raster sourced from the SRTM website (http://srtm.csi.cgiar.org/srtmdata/) and was created using ArcGIS software. The species photograph was provided by Anirban Chaudhuri through personal communication and were used with their prior permission.

**Fig 3:**
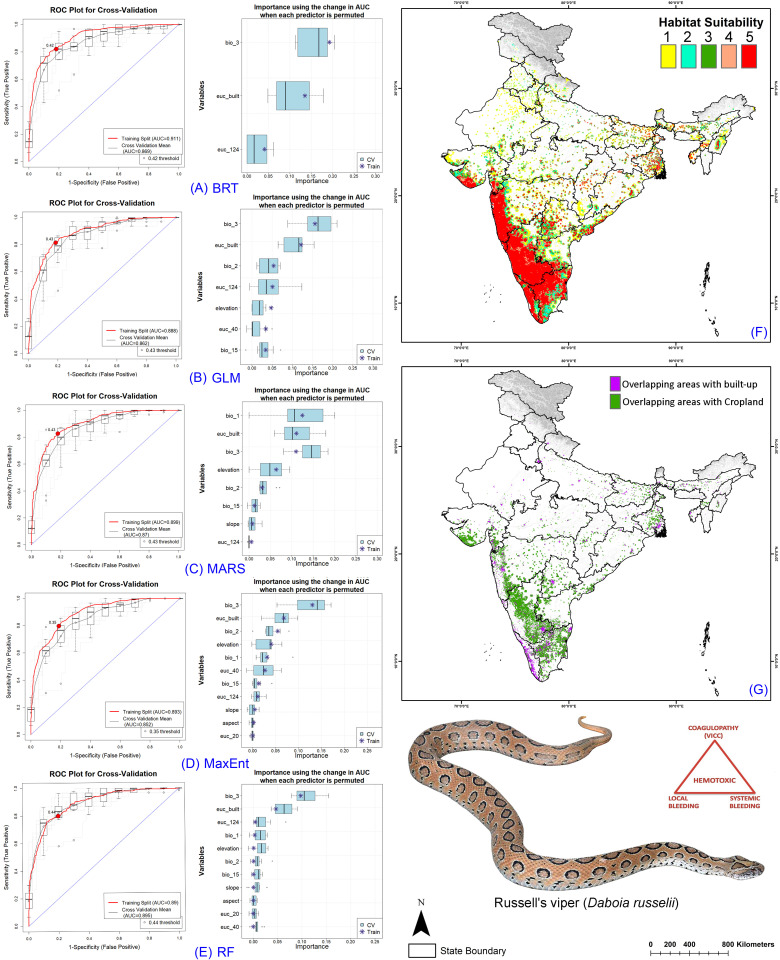
Model evaluation plot, showing the average training ROC of both training and cross-validation (CV) and variables selected by the models for the replicate runs under five models of *D. russelii.* (A) showing ROC plot of boosted regression tree (BRT), (B) generalized linear model (GLM), (C) multivariate adaptive regression spines (MARS), **(D)** Maximum entropy (MaxEnt), **(E)** Random Forest (RF), **(F)** The suitable habitat extent in the present scenario where the ‘class 5’ determines the extremely suitable habitat extent, and **(G)** The overlapping cropland and built-up/urban areas with the suitable extent (Class 4 and Class 5) in the present scenario. The administrative layer of the map was obtained from the DIVA-GIS website (https://diva-gis.org/data.html). The base layer of the maps is an elevation raster sourced from the SRTM website (http://srtm.csi.cgiar.org/srtmdata/) and was created using ArcGIS software. The species photograph was provided by Anirban Chaudhuri through personal communication and were used with their prior permission.

**Fig 4:**
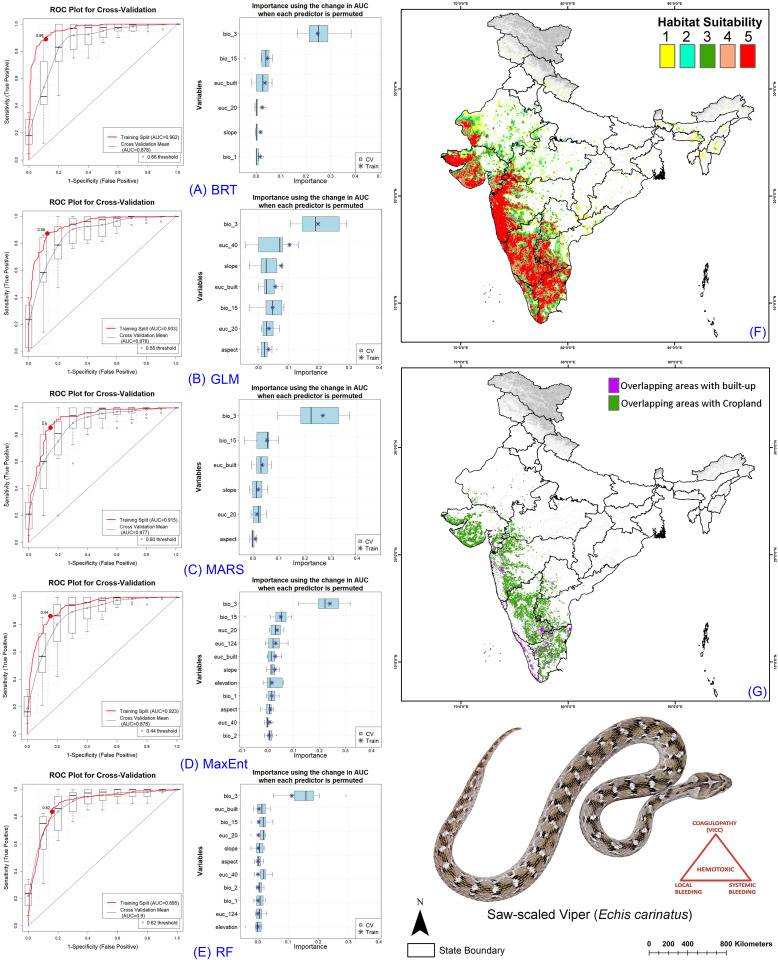
Model evaluation plot, showing the average training ROC of both training and cross-validation (CV) and variables selected by the models for the replicate runs under five models of *E. carinatus.* (A) showing ROC plot of boosted regression tree (BRT), (B) generalized linear model (GLM), (C) multivariate adaptive regression spines (MARS), **(D)** Maximum entropy (MaxEnt), **(E)** Random Forest (RF), **(F)** The suitable habitat extent in the present scenario where the ‘class 5’ determines the extremely suitable habitat extent, and **(G)** The overlapping cropland and built-up/urban areas with the suitable extent (Class 4 and Class 5) in the present scenario. The administrative layer of the map was obtained from the DIVA-GIS website (https://diva-gis.org/data.html). The base layer of the maps is an elevation raster sourced from the SRTM website (http://srtm.csi.cgiar.org/srtmdata/) and was created using ArcGIS software. The species photograph was provided by Raju Vyas through personal communication and were used with their prior permission.

**Fig 5:**
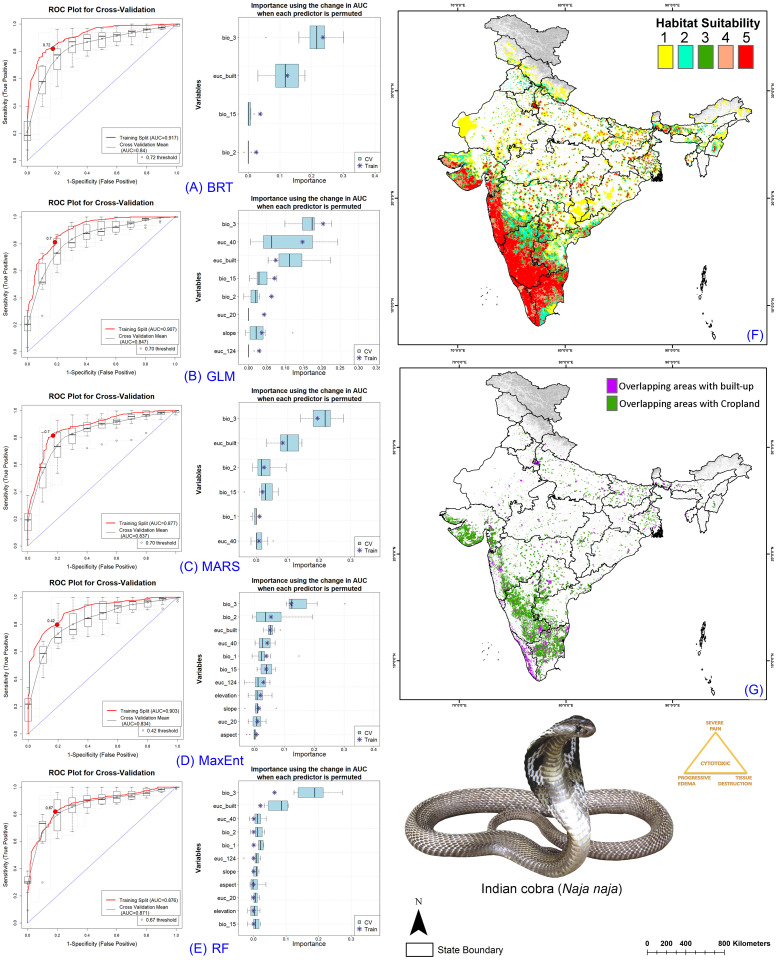
Model evaluation plot, showing the average training ROC of both training and cross-validation (CV) and variables selected by the models for the replicate runs under five models of *N. naja.* (A) showing ROC plot of boosted regression tree (BRT), (B) generalized linear model (GLM), (C) multivariate adaptive regression spines (MARS), **(D)** Maximum entropy (MaxEnt), **(E)** Random Forest (RF), **(F)** The suitable habitat extent in the present scenario where the ‘class 5’ determines the extremely suitable habitat extent, and **(G)** The overlapping cropland and built-up/urban areas with the suitable extent (Class 4 and Class 5) in the present scenario. The administrative layer of the map was obtained from the DIVA-GIS website (https://diva-gis.org/data.html). The base layer of the maps is an elevation raster sourced from the SRTM website (http://srtm.csi.cgiar.org/srtmdata/) and was created using ArcGIS software. The species photograph was provided by Anirban Chaudhuri through personal communication and were used with their prior permission.

The resulting model performance demonstrated excellence across all participating models, showing strong performance on both training and cross-validation datasets (Tables 1, S2 Figs 2–5, S7–S14). The models exhibited AUC values ranging from 0.866 to 0.962 during training and between 0.834 and 0.895 at cross-validation across the four species. Specifically, the lowest ΔAUC value was observed in the RF model for *E. carinatus*, while the highest ΔAUC value was seen in the BRT model for *N. naja*, with a value of 0.77. In all species runs, the RF model demonstrated the lowest ΔAUC value for each species. However, MaxEnt showed the highest ΔAUC value for three species, except for *E. carinatus*, where GLM demonstrated the highest value (Figs 2–5). Among the five selected models, MaxEnt utilized all variables provided in each replicate run, while BRT employed the fewest variables for the four species. Specifically, within the BRT models, the highest number of variables used was six out of 11 for *E. carinatus*, whereas the lowest number of variables selected was three out of 11 for *D. russelii* (Tables 1, S2 Figs 2–5).

**Table 1:** The ensembled model fit metrics for the five participating modelling methods and for the final ensemble model for estimation of habitat suitability of the Big Four species. A total of five model algorithms were used with the threshold of < 0.75 AUC score. AUC: Area under Curve, ΔAUC: Change in Area under curve (Training – Cross Validation), PCC: Proportion Correctly Classified, TSS: True Skill Statistic.

Species	Ensemble AUC	Ensemble PCC	Ensemble TSS	Ensemble Kappa	Ensemble Specificity	Ensemble Sensitivity
*B. caeruleus*	0.8516	78.80	0.5506	0.5250	0.7414	0.8094
*D. russelii*	0.8696	79.42	0.5856	0.5834	0.8078	0.7781
*E. carinatus*	0.8822	82.82	0.6522	0.6520	0.8132	0.8390
*N. naja*	0.8458	79.64	0.5634	0.5352	0.7470	0.8166

The ensemble model for the Big Four species indicated that, on average across the five models, the bioclimatic variable isothermality (BIO2/BIO7) (×100) (bio_3) was the highest contributor for all four species, accounting for more than 30% of the covariate contribution for each species (Tables 2, S3, S15–S18 Figs). Specifically, for *B. caeruleus*, the lowest contributing variable was slope, contributing only 0.29%. The habitat variable Euclidean Distance to Tree Cover (euc_124) contributed 9.2%, while Euclidean Distance to Built-up Areas was the primary anthropogenic contributor, accounting for 21.92%. For *D. russelii*, the least contributing variable was the topographic variable aspect, contributing 0.36%. The habitat variable Euclidean Distance to Tree Cover (euc_124) contributed 7.63%, and Euclidean Distance to Built-up Areas was once again the primary anthropogenic contributor, accounting for 20.57%. Similarly, in the case of *E. carinatus*, the least contributing variable was the bioclimatic variable Mean diurnal range (bio_2), accounting for 0.35%. Specifically, for *N. naja*, the least contributing variable was the topographic variable aspect, contributing 0.33%. Notably, the habitat variable Euclidean Distance to Tree Cover (euc_124) had relatively low contributions for both species, with 0.98% for *E. carinatus* and 3.44% for *N. naja*. Among anthropogenic variables, Euclidean distance to built-up areas was again the primary contributor, accounting for 9.15% for *E. carinatus* and 17.95% for *N. naja*. Additionally, for *E. carinatus*, the habitat variable Euclidean Distance to Rangeland/Bareland was a significant contributor, accounting for 6.5% overall (Tables 2, S3, S15–S18 Figs).

**Table 2:** Summary of the highest contributing variable from each category for each snake species, along with its mean (μ) percentage contribution to the model. This table highlights the most influential predictors across categories, while the full list of all final covariates and their contributions for the Big Four species is provided in S3 Table.

Species	Covariate Set	Variable	μ(Mean) %
*B. caeruleus*	Bioclimatic	Isothermality	42.59
	Habitat	Euclidean distance to Tree Cover	9.20
	Anthropogenic	Euclidean distance to Built-up/Urban	21.92
	Topographic	Elevation	0.67
*D. russelii*	Bioclimatic	Isothermality	32.32
	Habitat	Euclidean distance to Tree Cover	7.63
	Anthropogenic	Euclidean distance to Built-up/Urban	20.57
	Topographic	Elevation	3.21
*E. carinatus*	Bioclimatic	Isothermality	50.69
	Habitat	Euclidean distance to Barelands/Rangelands	6.50
	Anthropogenic	Euclidean distance to Built-up/Urban	9.15
	Topographic	Slope	8.60
*N. naja*	Bioclimatic	Isothermality	39.97
	Habitat	Euclidean distance to Tree Cover	3.44
	Anthropogenic	Euclidean distance to Built-up/Urban	17.95
	Topographic	Slope	2.67

### Identification of Habitat Extent of Big Four Species

The ensemble model revealed the extremely suitable habitat (class 5) extents in India for four species in the present scenario: *B. caeruleus* (124,497 sq. km.), *D. russelii* (88,740 sq. km.), *E. carinatus* (81,590 sq. km.), and *N. naja* (88,853 sq. km.) (Figs 2-5F, S4–S7 Tables). However, considering future climate change scenarios, the results indicate a significant shift in geographic range, with suitable habitats shrinking due to climatic changes. This shift raises concerns as many new states might experience increased snakebite cases in areas previously unaffected by this health issue (S19–S22 Figs, S4–S7 Tables). Specifically, *B. caeruleus* is expected to experience a decline of over 50% in a suitable extent, whereas *D. russelii* is projected to decline by approximately 70% to 80%, *E. carinatus* by 43% to 73%, and *N. naja* by 50% to 71%. The Indian state of Karnataka emerged as a hotspot for extremely suitable habitats for the Big Four in the present scenario, harboring the highest suitable habitat extent for each species. Conversely, some mainland states and Union Territories, such as Sikkim and Ladakh, were found to be unsuitable for the Big Four species.

In future climatic scenarios, the Big Four snake species are projected to experience a decline and shift in their geographic ranges, primarily towards Northern and Northeastern India (S19–S22 Figs, S4–S7 Tables). Notably, certain states currently identified as key hotspots such as Karnataka and Tamil Nadu, which presently exhibit the highest levels of habitat suitability are expected to experience a pronounced reduction in regions classified as highly suitable for these species. Interestingly in the case of *E*. *carinatus*, Karnataka presently boasts the most extensive coverage of class 5 habitats (indicating maximum suitability) is projected to experience the least severe reductions especially in the western region, with anticipated declines ranging between 12% and 44%. Contrastingly, specific regions including Haryana, Rajasthan, Assam etc. are projected to witness an expansion in suitable habitat extent in response to shifting climate conditions. Furthermore, states such as Manipur, Meghalaya, Nagaland, and Arunachal Pradesh, which currently lack any suitable habitat, are predicted to experience notable increases in habitat suitability for the Big Four in future climate scenarios. These Northeastern states are projected to experience a significant increase (>100%) in habitat suitability for the Big Four due to the anticipated changes in climatic conditions.

### Snakebite Prone Areas in Croplands and Built-up

In the current scenario, an analysis of the overlapping areas of cropland and built-up regions with habitats suitable for the Big Four species reveals a number of significant risk zones or hotspots across various states in India (Figs 2–5, S8–S15 Tables). Notably, the central region of Karnataka, Tamil Nadu, and Kerala in Southern India show the highest levels of overlap with cropland areas, highlighting these regions as critical areas of concern for public health safety. Additionally, certain areas in the western parts of Gujarat and Maharashtra also exhibit significant overlap with cropland. Moreover, these states also exhibit considerable overlap with human settlements, further amplifying the risk for envenomation. This pattern of overlap with anthropogenic landscapes is consistent across all four species in the present climate scenario.

However, as the geographic range of suitable habitats for the Big Four species shifts due to projected climate change, there is also a corresponding shift in the areas where these habitats overlap with cropland and built-up regions (S23-S26 Figs). In particular, southern and western states are expected to see a decline in the extent of overlap with human-modified landscapes as suitable habitats move northward and northeastward. This shift is a direct result of changing climate patterns, which are driving the species toward new regions. At the same time, northern and northeastern states are expected to witness a marked increase in the overlap of suitable habitats with built-up areas. This is particularly evident in regions such as Punjab, Uttar Pradesh, and West Bengal and is projected to become significant (Figs 2–5, S8–S15 Tables). These states, which have relatively low levels of overlap in the current scenario, will face growing challenges as urban and agricultural expansion coincides with an increase in suitable habitats for the Big Four species. Furthermore, other Northeastern states such as Assam, Meghalaya, and Nagaland are anticipated to experience a notable rise in the overlap of the Big Four species habitats with anthropogenic landscapes. This is a concerning trend, as it suggests that previously unaffected regions may soon become hotspots for human-snake interactions, posing challenges for public safety.

### Prioritization of big four bite prone regions in India

**Fig 6:**
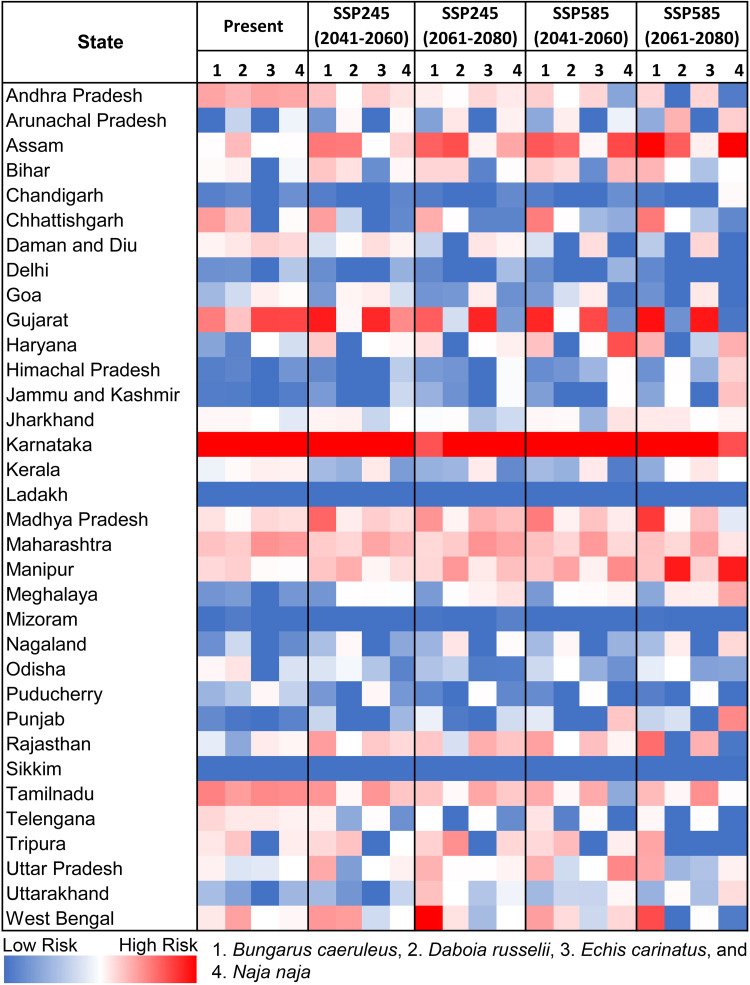
This figure illustrates the variation in risk indices of Big Four species across different Indian states, with color coding to distinguish risk levels in present and future climatic scenarios.

**Fig 7:**
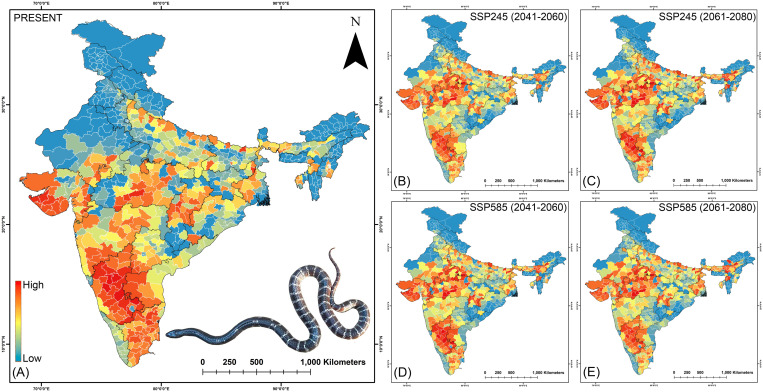
The maps indicate the risk index scores for prioritization of each district in the (A) present, (B) SSP245 (2041-2060), (C)SSP245 (2061-2080), (D) SSP585 (2041-2060), and (E) SSP585 (2061-2080) future scenarios for *B. caeruleus.* The administrative layer of the map was obtained from the DIVA-GIS website (https://diva-gis.org/data.html). The species photograph was provided by Anirban Chaudhuri through personal communication and were used with their prior permission.

**Fig 8:**
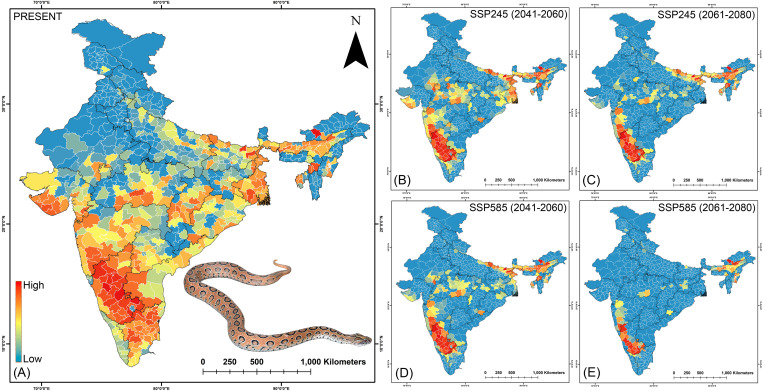
The maps indicate the risk index scores for prioritization of each district in the (A) present, (B) SSP245 (2041-2060), (C)SSP245 (2061-2080), (D) SSP585 (2041-2060), and (E) SSP585 (2061-2080) future scenarios for *D. russelii.* The administrative layer of the map was obtained from the DIVA-GIS website (https://diva-gis.org/data.html). The species photograph was provided by Anirban Chaudhuri through personal communication and were used with their prior permission.

In the case of *B. caeruleus*, the comprehensive risk assessment in the present scenario highlights that Karnataka holds the highest risk index score of 171.85 (Figs 6, 7, S16 Table). This score positions Karnataka as the most critical state followed by Gujarat (96.70) and Tamil Nadu (94.58), requiring immediate attention for snakebite mortality incidents caused by *B. caeruleus* (Figs 6,7). Furthermore, at the district level, the highest priority districts in the current scenario are Chikkaballapura (719.73), Haveri (693.56), and Chitradurga (674.78), all located in the state of Karnataka. In contrast, districts in the northern states such as Jammu & Kashmir and Ladakh, as well as those in the Northeastern region, currently exhibit comparatively lower risk index scores. The future climate change projections suggest a substantial geographic shift in the habitat suitability for *B. caeruleus*, which in turn affects the risk index scores across various states (Figs 6,7). Notably, states such as Gujarat (with up to a 73.61% increase), Madhya Pradesh (ranging between 224.18% and 308.25%), and Assam (reaching as high as 783.24%) are projected to witness a sharp rise in risk scores, particularly under extreme climatic conditions during the 2061–2080 period. At the district level, areas such as Datia (Madhya Pradesh), Mahoba in Uttar Pradesh, and Karbi Anglong West, Golaghat, and Nagaon (Assam) are expected to experience the most significant increases in risk index scores under future climate scenarios (Figs 6,8, S16 Table). A similar trend is evident for *D. russelii*, with Karnataka once again emerging as the highest priority state, exhibiting a risk index of 150.38 in the current scenario (Fig 8, S17 Table). It is followed by Tamil Nadu and West Bengal, with risk index values of 66.13 and 63.43, respectively. Notably, East Kameng district in Arunachal Pradesh stands out as a major outlier, exhibiting the highest district-level risk index of 729.99, likely attributable to significant socio-economic deprivation, i.e., GRDI of the region. Following East Kameng, the high-priority districts include Chikkaballapura, Haveri, and Chitradurga in Karnataka, along with Yanam in Andhra Pradesh (Fig 8). In future climatic scenarios, Karnataka consistently remains a top-priority state across all climate projections and time periods. Notably, Assam emerges as the second-highest priority state, followed by Manipur, with both states experiencing a risk index increase of over 6%. At the district level, Haveri, Ramanagara, and Chamarajanagar, located in Karnataka, are projected to be among the highest priority areas. Additionally, districts such as Morigaon, Nagaon, and Lakhimpur in Assam, Pratapgarh in Rajasthan, and Supaul in Bihar are expected to witness more than a two-fold increase in risk index scores under future climatic conditions, although they still rank below the highest-risk districts. In contrast, the East Kameng district in Arunachal Pradesh is projected to experience a significant decline of over 50% in its risk index in future scenarios (Fig 8, S17 Table).

**Fig 9:**
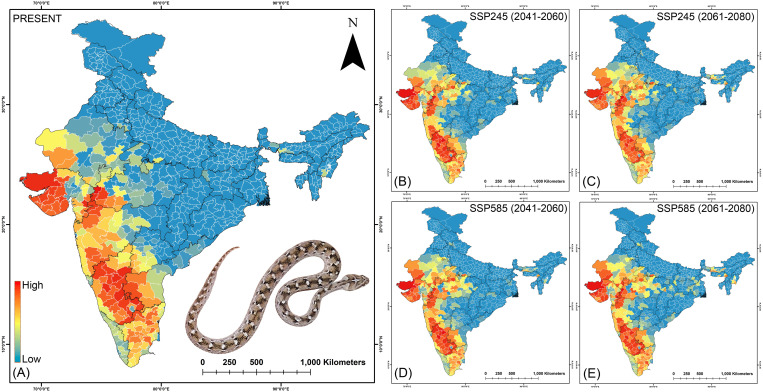
The maps indicate the risk index scores for prioritization of each district in the (A) present, (B) SSP245 (2041-2060), (C)SSP245 (2061-2080), (D) SSP585 (2041-2060), and (E) SSP585 (2061-2080) future scenarios for *E. carinatus.* The administrative layer of the map was obtained from the DIVA-GIS website (https://diva-gis.org/data.html). The species photograph was provided by Raju Vyas through personal communication and were used with their prior permission.

**Fig 10:**
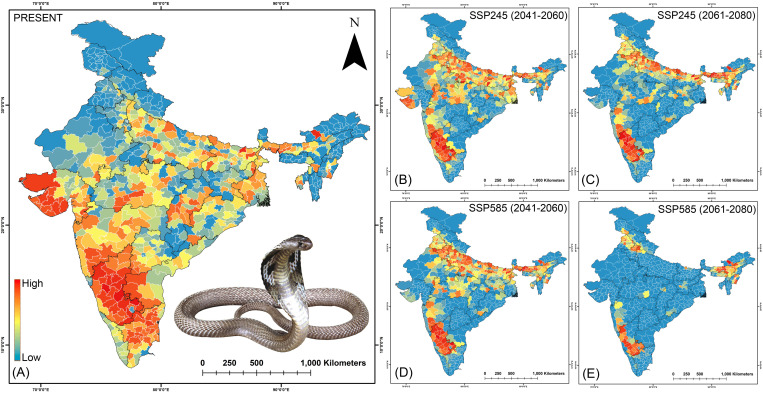
The maps indicate the risk index scores for prioritization of each district in the (A) present, (B) SSP245 (2041-2060), (C)SSP245 (2061-2080), (D) SSP585 (2041-2060), and (E) SSP585 (2061-2080) future scenarios for *N. naja.* The administrative layer of the map was obtained from the DIVA-GIS website (https://diva-gis.org/data.html). The species photograph was provided by Anirban Chaudhuri through personal communication and were used with their prior permission.

The state of Karnataka, with a risk index of 135.02, remains the highest priority state for *E. carinatus* also, followed by Gujarat (99.47) and Tamil Nadu (63.27) under current climatic conditions (Figs 6, 9, S18 Table). While within the state at the district level, Jhabua (Madhya Pradesh), Koppal, and Chikkaballapura in Karnataka are identified as the most vulnerable districts in the present scenario. The projections under future climatic conditions indicate a decline in risk index scores for these currently high-priority states, as Karnataka is expected to experience a reduction of over 18%, Gujarat (ranging between 6.07% and 21.88%), and Tamil Nadu (up to 37.06%). Conversely, Rajasthan and Madhya Pradesh are projected to exhibit substantial increases in risk index values, rising by as much as 227.84% and 61.11%, respectively. While Jhabua, Koppal, and Chikkaballapura are projected to see a decline exceeding 15.61%, they are anticipated to remain among the highest priority districts relative to others. Notably, districts such as Ashoknagar (Madhya Pradesh) and Pratapgarh (Rajasthan) are projected to exhibit significant increases in their risk index values, approximately doubling relative to current levels under future climatic scenarios (Fig 9, S18 Table). However, despite this sharp rise, they are expected to remain below the highest-risk districts in terms of the risk index. Meanwhile For *N. naja*, Karnataka and Gujarat once again emerge as top-priority states in the current scenario, with risk index values of 135.67 and 100.41, respectively (Figs 6,10, S19 Table). This pattern is reflected at the district level as well, with Chikkaballapura, Chitradurga, and Haveri, all located within the state of Karnataka, having been identified as the highest-risk districts under present conditions. However, under future climatic scenarios, these high-priority states show a marked decline in risk index values. Specifically, Karnataka, Maharasthra, Gujarat, and Tamil Nadu are projected to experience reductions exceeding 16%, with Gujarat showing the most pronounced decline of over 42.40%, likely driven by climatic changes. Conversely, states such as Assam, Rajasthan, and Manipur are expected to see substantial increases in future risk, with some projections indicating more than a doubling of their current risk index values. At the district level, the trends are more dynamic as for instance, Chikkaballapura and Haveri are projected to experience an increase in risk index during the 2041–2060 period under both SSP scenarios, followed by a sharp decline during the 2061–2080 period across both emission pathways. Meanwhile, districts such as Tengnoupal (Manipur), Sirsa, and Mewat in Haryana are projected to exhibit significant increases in vulnerability, with substantial rises in risk index scores under future climate projections (Fig 10, S19 Table). These findings highlight the urgent need for targeted intervention and strategic planning in these states and districts to address snakebite-related mortality and morbidity in the face of evolving climate risks. The sensitivity analysis of the Risk Index at both district and state levels revealed that *B. caeruleus* exhibited the highest sensitivity among the Big Four species, consistently showing the highest Area Under the Curve (AUC) values across all future climate projections (S27-S30 Figs). In contrast, *D. russelii* was identified as the least sensitive species at both district and state levels, as it consistently displayed the lowest AUC values across all climate scenarios.

## Discussion

Snakebite, a neglected tropical disease (NTD), continues to affect large numbers of people, disproportionately impacting vulnerable populations and regions throughout the globe [76]. Specifically, several South Asian countries are projected to become more climatically suitable for venomous snakes by 2070, potentially altering the distribution of venomous species as they migrate to new locations as evidenced hence increasing conflict with human [14,77–79]. Consequently, the previous studies have emphasized the need for enhanced surveillance and monitoring to identify prone areas of NTD incursion and emergence, ensuring their inclusion in future public health strategies [76]. Furthermore, snake envenomation in India poses a significant challenge for antivenom manufacturers due to the necessity for highly region-specific production and subsequent treatments [8,15,78]. The venom composition within a single species may vary significantly due to several factors such as age, gender, prey availability, diet, and geographic location [80–82]. Hence, identifying suitable habitats and delineating high-risk areas for snakebites under current and future climatic conditions might be effective for snakebite management and the targeted allocation of region-specific antivenoms, thereby aiding public health [35,83,84]. This forward-looking approach that incorporates future climate scenarios is essential for mitigating potential risks and enhancing snakebite management strategies to address forthcoming NTD challenges.

The future climatic projections of the present study indicate a geographical shift of the Big Four species towards the Northern and Northeastern states of India aligning with previous zoo-geographic studies [14]. Furthermore, Northeast India and Southeast Asia have historically supported high snake diversity due to their position within transitional zones between major eco-regions, functioning as climatic refugia [85]. As global temperatures continue to rise, the shifts in species ranges including both contractions and expansions, are increasingly expected to occur in regions with long-established biodiversity and complex, heterogeneous landscapes [85]. In light of this, the ensemble model identified Isothermality (bio_3) as the most significant bioclimatic factor contributing to the habitat suitability for the Big Four species. This variable quantifies the extent to which day-to-night temperature oscillations compare to the larger annual summer-to-winter temperature fluctuations. These climatic variables were also found to align with previous studies on congeners of the Big Four snake species [39]. The substantial influence of temperature fluctuations on the distribution of cold-blooded species has likewise been corroborated by earlier research [14,77]. The climatic parameters are often the most influential predictors of snake distributions due to their significant effects on the thermal biology, activity patterns, and reproductive success of ectothermic organisms [86–88]. These broad-scale environmental filters shape ecological niches more profoundly than land use variables, particularly for thermally sensitive taxa like snakes [89]. Nevertheless, integrating both climatic and land use variables is recommended for more reliable and holistic predictions of habitat suitability [70]. Furthermore, the ecological model revealed that the South Indian states, including Karnataka, Kerala, and Tamil Nadu, particularly the Western Ghats region, are hotspots for the Big Four species in the present scenario, thus aligning with previous studies conducted in the region [90,91]. Moreover, considering that the majority of snakebites attributed to the Big Four species occur in agricultural or settlement areas, it becomes imperative to identify regions of overlapping cropland and built-up areas with suitable habitats for these species within the Indian states [92,93] The thorough understanding of these overlap zones is essential for effective health management, which enables the implementation of preventive measures and improvement of treatment responses, as emphasized by previous research [14]. The current study identifies the susceptibility of South Indian states under present conditions and projects an increase in vulnerability driven by anticipated climate change within the Northern and Northeastern states in the future. However, the state of Assam, in particular, stands out due to a consistent rise in snakebite related mortality and morbidity, also evidenced by recent sightings and increased reporting of species like *D. russelii* and *N. naja*, disseminated widely through social and news media channels. This may be attributed to the fact that many tropical snake species have an affinity for tropical, forested lowlands with a tropical climate and abundant water bodies, a characteristic that is particularly prominent in Assam, given its lowland geography compared to other Northeast Indian states [94–97]. These findings align closely with the study’s projections of escalating suitability and habitat overlap in future climatic conditions of Assam. However, states such as Meghalaya, Manipur, and Nagaland also exhibit increased overlap potential for the Big Four species, as these states are known to be providing suitable habitats for other congeners. Additionally, this observed pattern may be indicative of an altitudinal shift in the distribution of snake species, likely driven by climate-induced changes in temperature and habitat conditions. Such vertical range shifts have been widely documented among ectothermic taxa, including reptiles, as they track suitable thermal environments to higher elevations in response to warming climate [98,99]. While this suggests a potential ecological response to climate stressors, it is important to note that the overall habitat suitability in mountainous regions remain relatively lower compared to the lowland plains. This discrepancy may be attributed to factors such as limited thermal buffering capacity at higher altitudes, reduced availability of optimal prey and shelter, etc. Furthermore, this aspect also raises concerns about the inadvertent introduction of Big Four species through activities like timber transportation, smuggling, and accidental or deliberate releases, particularly in the northeastern states, which are key corridors for illegal wildlife trafficking [100]. The anticipated suitability of these regions under future climatic conditions may exacerbate the risk of population surges among these species, especially when both sexes have been exposed through human mediated migration.

Moreover, the risk index identified priority states such as Karnataka, Andhra Pradesh, Gujarat, etc. that require the utmost attention to reduce mortality from bites by the Big Four species. These states are high-risk areas due to their significant overlap extent along with socioeconomic deprivation and the available government infrastructure. This finding aligns with previous studies that reported the highest number of deaths from Big Four species bites in the past decade from these states [15]. Interestingly, Tamil Nadu ranks relatively low on the risk index despite being a hotspot for the Big Four species and having a similar extent of overlap with cropland and built-up areas as Karnataka [101]. Although Tamil Nadu experiences a significantly higher number of snakebite incidents, the discrepancy in the risk index can be attributed to its low GRDI and relatively higher number of healthcare facilities compared to Karnataka [101]. These factors help reduce the likelihood of fatalities from snakebite envenomation, leading to a lower overall risk index than that of Karnataka. Similarly, Sikkim presents an interesting case, as it is classified as a state with no risk from the Big Four snake species. While some congeners of these species, such as *B. niger, N. kaouthia*, etc., are documented in the southern districts of Sikkim, the Big Four have not yet been officially reported in the state [102]. An SDM study also identified suitable habitats for these congeners in the same regions [102]. Consequently, the vulnerability in Sikkim was considered low, as the present study focused specifically on the Big Four species, which remain unreported in the state. Nonetheless, there may still be potential risk from their congeners, which needs to be studied further. Moreover, the high GRDI and low healthcare infrastructure in East Kameng District of Arunachal Pradesh likely explain why it stands as an outlier for *D. russelii*. Despite this district having a much lower overlap with cropland and built-up areas, East Kameng recorded the highest risk index for *D. russelii* in present scenario, highlighting the significant impact of healthcare access and disease management in determining risk levels. This observed association between higher GRDI and increased snakebite risk can be attributed to overlapping vulnerabilities such as poor healthcare access, low socioeconomic conditions, and greater exposure to high-risk environments. The empirical studies have shown that snakebite morbidity and mortality are disproportionately higher in impoverished, rural areas where timely medical intervention is often lacking and human-wildlife interactions are more frequent [101,103]. These findings support the role of GRDI as a proxy indicator for identifying populations that may face elevated snakebite risk due to structural and environmental disadvantages. Additionally, the sensitivity analysis corroborates that *B. caeruleus* exhibits the highest sensitivity to climate change among the four species, both at the district and state levels. This species shows notable proliferation under various future climate scenarios compared to the present. In contrast, *D. russelii* appears to be the least sensitive, as it shows an overall decline in range across all future climate scenarios. However, it exhibits a spatial shift, becoming concentrated in biodiversity hotspots such as the Western Ghats and Northeast India. A similar pattern is observed in *N. naja*, which also shows future range contractions with concentration in the Western Ghats and the Himalayan region. Meanwhile, *E. carinatus* remains predominantly distributed in the western parts of India, likely due to its preference for arid and semi-arid conditions. These projections suggest that, in addition to climatic factors, environmental parameters significantly influence the distribution patterns of the Big Four snake species [40–43]. Consequently, states in southern India (e.g., Karnataka, Gujarat, Andhra Pradesh) and in northern and northeastern regions (e.g., Rajasthan, Haryana, Assam, Manipur) must adopt proactive conservation and mitigation strategies. Moreover, many of the Indian states are experiencing significant habitat destruction of Big Four species, which may result in increased human-snake conflicts as they tend to move for prey near human settlements [14,104]. Therefore, addressing the issue of envenomation requires a multifaceted and coordinated approach involving various stakeholders, including international agencies, government bodies, non-governmental organizations, and local communities. Several critical steps must be taken to mitigate envenomation incidents, or at least manage them effectively when they occur. First and foremost, continuous education on snake identification, envenomation, and the application of preventive first aid at both rural and urban areas are crucial in the detected high priority districts such as Chikkaballapura, Haveri, and Chitradurga etc. in Karnataka; Devbhumi Dwarka and Jamnagar etc. in Gujarat; Pratapgarh in Rajasthan; Nagaon, Morigaon, and Golaghat etc. in Assam; East Kameng in Arunachal Pradesh, and Tengnoupal in Manipur etc. [8,15]. Such education should not be a one-time effort but a continuous process to ensure that all age groups are equally aware of the risks and appropriate responses to snakebites. Furthermore, raising awareness among local peoples about the importance of seeking medical treatment from hospitals rather than turning to exorcists or faith healers is also essential. This type of awareness education is especially crucial in the tribal dominated regions of Southern India, Central India, and the Northeastern states, where faith healers hold significant influence within tribal communities. Moreover, rural hospitals and primary healthcare facilities within these priority regions need to be strengthened by increasing their capacity and ensuring they are staffed with trained personnel capable of effectively managing snakebite cases. Furthermore, ensuring the consistent availability of sufficient quantities of antivenom in these areas is critical to reducing snakebite-related mortality. This will enable to prevent deaths during the time taken to travel to sophisticated government health facilities in urban areas. As it is evident that, the effectiveness of antivenoms in reducing mortality by over 90% when administered promptly [8,15,35,84,105–109]. This targeted attention is particularly important for the hilly terrains of the Northeastern region, especially in remote areas such as East Kameng in Arunachal Pradesh, Karbi Anglong in Assam, and Tengnoupal in Manipur, where connectivity to remote villages is often limited. Additionally, the production of region-specific monovalent antivenoms is necessary to ensure the highest efficacy against locally prevalent population of Big Four species. Along with the production of antivenoms, it is also essential to maintain a robust supply chain and ensure that antivenoms are distributed to all remote regions, especially to the identified high priority states and districts in India. Additionally, based on current biogeographic and risk indexing, the establishment of rapid response units in both urban and rural areas of snakebite-prone districts are recommended. These units would coordinate with the kin of the victim to provide first aid and ensure quick access to the nearest medical facility for timely treatment. Furthermore, implementing an online reporting system to monitor antivenom distribution and track snakebite cases would be a valuable tool, enabling real-time data collection to inform and strengthen targeted response efforts. Moreover, the response unit could also be tasked with rescuing snakes from high-priority areas, thereby reducing the risk of snake envenomation incidents and minimizing retaliatory killings of these reptiles. In addition to these interventions, it is necessary to conduct ecological studies and regular monitoring of the Big Four species in districts or states where the Risk Index is projected to decline, to better understand potential ecological adaptations or the persistence of micro-habitats supporting these populations. Notably, studies of this nature should be periodically replicated to incorporate updated environmental and socioeconomic variables reflective of the prevailing conditions. Such replication ensures that risk assessments remain relevant and effective at the time of implementation by accounting for dynamic changes like land cover alterations, thereby enabling more precise and timely interventions to combat this NTD.

This study stands as the first comprehensive ecological analysis targeting snakebite hotspots in India, specifically focusing on the ecological dynamics of the Big Four species under both current and future climatic scenarios. The holistic methodology of a detailed risk index that identifies states and districts in urgent need of intervention from policymakers and health planners, highlighting high-risk areas and guiding resource allocation to effectively mitigate snakebite risks presently and in the future. Therefore, by identifying regions in need of immediate attention, the study helps prioritize states and districts for improving healthcare infrastructure and ensuring adequate antivenom supply, crucial for addressing current snakebite challenges and preparing for future risks amplified by climate change. Furthermore, this study will support the WHO and its partners in implementing the 2019 Snakebite Envenoming Strategy for prevention and control in snakebite-prone countries for planning and policy integration. However, the results and interpretations require ground validation by expert personnel to ensure effective application in the field. Additionally, rapid anthropogenic expansion and potential natural calamities impacting the micro-habitats of the Big Four snake species must be taken into account alongside the inferences from this study. Such considerations are crucial for future policy-making, as this research provides a preliminary indication of possible trends and challenges that may affect these species habitats over time. Moreover, future research could incorporate dynamic socio-economic modeling to capture potential shifts in land use patterns and developmental initiatives for the Big Four and other reptilian fauna. Nevertheless, beyond the health implications, this study highlights areas needing focused efforts to reduce human-snake conflicts and to conserve these ecologically significant species.

## Limitations

There are several important limitations associated with this study, primarily related to the use of SDMs. These models generate probabilistic outputs that are highly dependent on the quality, completeness, and accuracy of input data. As such, minor changes in the input parameters may lead to slight variations in the results; however, the overall patterns and trends generally remain consistent. It is also important to note that SDMs identify areas with suitable environmental conditions based on the ecological envelope of the species, rather than confirming actual presence. Although the Big Four snake species have broad geographic ranges across India, verified occurrence records are heavily concentrated in the southern and western regions due to frequent sightings. In contrast, certain areas in the northern and northeastern states show limited or no documented sightings, likely due to under reporting or the rarity of encounters. This discrepancy highlights a fundamental limitation of SDMs, which rely exclusively on presence-only data. As a result, regions lacking confirmed occurrence records must be excluded from the modeling process, as this can introduce spatial biases. The present study utilized current habitat layers for projecting future climatic suitability due to the unpredictability of future land cover changes. Moreover, the projections do not account for potential shifts in land use changes that may occur over time. The observed distributional changes in the species are therefore attributed solely to climatic variables. While this approach provides valuable insights into climate-driven range shifts, it may underestimate or overlook the compounded effects of land use change, urbanization, and habitat degradation. Therefore, future research should aim to incorporate land cover data that corresponds to the projected time periods alongside climatic variables. The use of the GRDI provided a consistent and nationally comparable measure of socioeconomic vulnerability, though future studies could enhance this with higher-resolution, region-specific data and associated uncertainty assessments. Despite these limitations, the approach presented here offers a novel framework that can be further refined and integrated into future research in a specific region by incorporating more comprehensive field surveys and citizen science data. Future studies should focus on expanding occurrence datasets and validating predictions through ground-truthing and ecological monitoring to enhance the reliability of SDMs in guiding conservation and public health interventions.

## Conclusion

Snake envenomation is a significant medical emergence in India, disproportionately affecting the economically deprived. Numerous studies have identified India as the most severely impacted country globally, with the highest number of snakebite fatalities, primarily attributed to the Big Four species. Furthermore, previous research has predominantly aimed at reducing fatalities or envenomations within the country. Species distribution models have been valuable in studying venomous snake distributions and assessing the impact of climate change on their range patterns. Notably, no prior research has integrated overlapping cropland and built-up areas with socioeconomic scenarios and healthcare infrastructure to prioritize Indian states and districts in present and future contexts, thereby understanding and quantifying their vulnerability to mortality by the Big Four species. This state and district-wise risk assessment is, to the knowledge of the authors, the first to predict the impact of climate change on the distribution of the Big Four species while highlighting regions at high risk from snakebite related mortality and morbidity. Furthermore, this study incorporates socioeconomic factors and healthcare infrastructure to prioritize Indian states and districts for urgent medical intervention by authorities based on both present and future projections. The findings will significantly assist the government, international agencies, and non-governmental organizations in prioritizing regions for targeted intervention for mitigating Big Four envenomation related mortality. The projected shift in the distribution range of the Big Four snake species in India is expected to be more pronounced towards the northern and northeastern states. This shift significantly overlaps with agricultural and urban areas, increasing the risk of snakebites in these regions in the near future. Addressing the challenges posed by these species as a result of climate change will require coordinated interventions from government authorities, regional and states and districts-level health organizations, and the international community to manage this NTD effectively in India.

## Supporting information

S1 Fig The maps represent the study area (India) and the IUCN distribution extent of Big Four species along with their occurrence points retrieved from the secondary sources: (A) *B. caeruleus*, (B) *D. russelii*, (C) *E. carinatus*, (D) *N. naja.* The administrative layer of the map was obtained from the DIVA-GIS website (https://diva-gis.org/data.html) and was created using ArcGIS software. The species photographs were provided by Anirban Chaudhuri and Raju Vyas through personal communication and were used with their prior permission.
(TIF)

S2 Fig Final set of variables retained for analysis after excluding highly correlated covariates. The figure illustrates the pairwise correlations (|r| < 0.8) among variables selected for *B. caeruleus*. The Pearson’s correlation coefficient is used as the primary measure. If either the Spearman or Kendall coefficient exceeds the Pearson value for a given pair, it is indicated with an “s” (Spearman) or “k” (Kendall) in the bottom-right corner of the corresponding cell.
(TIF)

S3 Fig Final set of variables retained for analysis after excluding highly correlated covariates. The figure illustrates the pairwise correlations (|r| < 0.8) among variables selected for *D. russelii*. The Pearson’s correlation coefficient is used as the primary measure. If either the Spearman or Kendall coefficient exceeds the Pearson value for a given pair, it is indicated with an “s” (Spearman) or “k” (Kendall) in the bottom-right corner of the corresponding cell.
(TIF)

S4 Fig Final set of variables retained for analysis after excluding highly correlated covariates. The figure illustrates the pairwise correlations (|r| < 0.8) among variables selected for *E. carinatus*. The Pearson’s correlation coefficient is used as the primary measure. If either the Spearman or Kendall coefficient exceeds the Pearson value for a given pair, it is indicated with an “s” (Spearman) or “k” (Kendall) in the bottom-right corner of the corresponding cell.
(TIF)

S5 Fig Final set of variables retained for analysis after excluding highly correlated covariates. The figure illustrates the pairwise correlations (|r| < 0.8) among variables selected for *N. naja*. The Pearson’s correlation coefficient is used as the primary measure. If either the Spearman or Kendall coefficient exceeds the Pearson value for a given pair, it is indicated with an “s” (Spearman) or “k” (Kendall) in the bottom-right corner of the corresponding cell.
(TIF)

S6 Fig The figure determines the Global Gridded Relative Deprivation Index (GRDI) v1 (2010–2020) obtained from NASA’s Socioeconomic Data and Applications Center (SEDAC) (https://cmr.earthdata.nasa.gov/). The administrative layer of the map was obtained from the DIVA-GIS website (https://diva-gis.org/data.html) and was created using ArcGIS software.
(TIF)

S7 Fig Confusion matrixes, Model Calibration plots and Residual plots for *B. caeruleus.* Row 1 represents spatial pattern of residuals where colour ramp indicates the magnitude of deviance and size represents the quantity. Row 2 represents model calibration plot across all 5 different model for cross-validation split. Row 3 represents confusion matrix for all 5 models, plotted by observed vs. predicted where colour ramp from lowest value 0% (white) to 100% (red) indicates the quantification of particular pair types. Column a. represents plots for BRT, Column b. represents plots for GLM, Column c. represents plots for MARS, Column d. represents plots for MaxEnt and Column e. represents plots for RF.
(TIF)

S8 Fig Confusion matrixes, Model Calibration plots and Residual plots for *D. russelii.* Row 1 represents spatial pattern of residuals where colour ramp indicates the magnitude of deviance and size represents the quantity. Row 2 represents model calibration plot across all 5 different model for cross-validation split. Row 3 represents confusion matrix for all 5 models, plotted by observed vs. predicted where colour ramp from lowest value 0% (white) to 100% (red) indicates the quantification of particular pair types. Column a. represents plots for BRT, Column b. represents plots for GLM, Column c. represents plots for MARS, Column d. represents plots for MaxEnt and Column e. represents plots for RF.
(TIF)

S9 Fig Confusion matrixes, Model Calibration plots and Residual plots for *E. carinatus.* Row 1 represents spatial pattern of residuals where colour ramp indicates the magnitude of deviance and size represents the quantity. Row 2 represents model calibration plot across all 5 different model for cross-validation split. Row 3 represents confusion matrix for all 5 models, plotted by observed vs. predicted where colour ramp from lowest value 0% (white) to 100% (red) indicates the quantification of particular pair types. Column a. represents plots for BRT, Column b. represents plots for GLM, Column c. represents plots for MARS, Column d. represents plots for MaxEnt and Column e. represents plots for RF.
(TIF)

S10 Fig Confusion matrixes, Model Calibration plots and Residual plots for *N. naja.* Row 1 represents spatial pattern of residuals where colour ramp indicates the magnitude of deviance and size represents the quantity. Row 2 represents model calibration plot across all 5 different model for cross-validation split. Row 3 represents confusion matrix for all 5 models, plotted by observed vs. predicted where colour ramp from lowest value 0% (white) to 100% (red) indicates the quantification of particular pair types. Column a. represents plots for BRT, Column b. represents plots for GLM, Column c. represents plots for MARS, Column d. represents plots for MaxEnt and Column e. represents plots for RF.
(TIF)

S11 Fig Evaluation Matrix performance across model runs for *B. caeruleus.* Brown - represents the correlation coefficient among the 5 different models. Yellow - represents the proportion of deviance explained; Green - represents the Proportion of correctly classified; Blue - represents Area under curve (AUC) and Pink - represents true skill statistics.
(TIF)

S12 Fig Evaluation Matrix performance across model runs for *D. russelii.* Brown - represents the correlation coefficient among the 5 different models. Yellow - represents the proportion of deviance explained; Green - represents the Proportion of correctly classified; Blue - represents Area under curve (AUC) and Pink - represents true skill statistics.
(TIF)

S13 Fig Evaluation Matrix performance across model runs for *E. carinatus.* Brown - represents the correlation coefficient among the 5 different models. Yellow - represents the proportion of deviance explained; Green - represents the Proportion of correctly classified; Blue - represents Area under curve (AUC) and Pink - represents true skill statistics.
(TIF)

S14 Fig Evaluation Matrix performance across model runs for *N. naja.* Brown - represents the correlation coefficient among the 5 different models. Yellow - represents the proportion of deviance explained; Green - represents the Proportion of correctly classified; Blue - represents Area under curve (AUC) and Pink - represents true skill statistics.
(TIF)

S15 Fig The response curves of the covariates selected by each of the participating ensemble model for *B. caeruleus.* (A) BRT, (B) GLM, (C) MARS, (D) MAXENT, and (E) RF.
(TIF)

S16 Fig The response curves of the covariates selected by each of the participating ensemble model for *D. russelii.* (A) BRT, (B) GLM, (C) MARS, (D) MAXENT, and (E) RF.
(TIF)

S17 Fig The response curves of the covariates selected by each of the participating ensemble model for *E. carinatus.* (A) BRT, (B) GLM, (C) MARS, (D) MAXENT, and (E) RF.
(TIF)

S18 Fig The response curves of the covariates selected by each of the participating ensemble model for *N. naja.* (A) BRT, (B) GLM, (C) MARS, (D) MAXENT, and (E) RF.
(TIF)

S19 Fig The maps represent the suitable habitat extent for *B. caeruleus* in the future SSP scenarios from the present scenario. Here the ‘class 5’ determines the extremely suitable habitat extent. The administrative layer of the map was obtained from the DIVA-GIS website (https://diva-gis.org/data.html). The base layer of the maps is an elevation raster sourced from the SRTM website (http://srtm.csi.cgiar.org/srtmdata/) and was created using ArcGIS software.
(TIF)

S20 Fig The maps represent the suitable habitat extent for *D. russelii* in the future SSP scenarios from the present scenario. Here the ‘class 5’ determines the extremely suitable habitat extent. The administrative layer of the map was obtained from the DIVA-GIS website (https://diva-gis.org/data.html). The base layer of the maps is an elevation raster sourced from the SRTM website (http://srtm.csi.cgiar.org/srtmdata/) and was created using ArcGIS software.
(TIF)

S21 Fig The maps represent the suitable habitat extent for *E. carinatus* in the future SSP scenarios from the present scenario. Here the ‘class 5’ determines the extremely suitable habitat extent. The administrative layer of the map was obtained from the DIVA-GIS website (https://diva-gis.org/data.html). The base layer of the maps is an elevation raster sourced from the SRTM website (http://srtm.csi.cgiar.org/srtmdata/) and was created using ArcGIS software.
(TIF)

S22 Fig The maps represent the suitable habitat extent for *N. naja* in the future SSP scenarios from the present scenario. Here the ‘class 5’ determines the extremely suitable habitat extent. The administrative layer of the map was obtained from the DIVA-GIS website (https://diva-gis.org/data.html). The base layer of the maps is an elevation raster sourced from the SRTM website (http://srtm.csi.cgiar.org/srtmdata/) and was created using ArcGIS software.
(TIF)

S23 Fig The maps represent the overlapping cropland and built-up/urban areas with the suitable extent (Class 4 and Class 5) for *B. caeruleus* in the future SSP scenarios from the present scenario. The administrative layer of the map was obtained from the DIVA-GIS website (https://diva-gis.org/data.html). The base layer of the maps is an elevation raster sourced from the SRTM website (http://srtm.csi.cgiar.org/srtmdata/) and was created using ArcGIS software.
(TIF)

S24 Fig The maps represent the overlapping cropland and built-up/urban areas with the suitable extent (Class 4 and Class 5) for *D. russelii* in the future SSP scenarios from the present scenario. The administrative layer of the map was obtained from the DIVA-GIS website (https://diva-gis.org/data.html). The base layer of the maps is an elevation raster sourced from the SRTM website (http://srtm.csi.cgiar.org/srtmdata/) and was created using ArcGIS software.
(TIF)

S25 Fig The maps represent the overlapping cropland and built-up/urban areas with the suitable extent (Class 4 and Class 5) for *E. carinatus* in the future SSP scenarios from the present scenario. The administrative layer of the map was obtained from the DIVA-GIS website (https://diva-gis.org/data.html). The base layer of the maps is an elevation raster sourced from the SRTM website (http://srtm.csi.cgiar.org/srtmdata/) and was created using ArcGIS software.
(TIF)

S26 Fig The maps represent the overlapping cropland and built-up/urban areas with the suitable extent (Class 4 and Class 5) for *N. naja* in the future SSP scenarios from the present scenario. The administrative layer of the map was obtained from the DIVA-GIS website (https://diva-gis.org/data.html). The base layer of the maps is an elevation raster sourced from the SRTM website (http://srtm.csi.cgiar.org/srtmdata/) and was created using ArcGIS software.
(TIF)

S27 Fig Receiver Operating Characteristic (ROC) curve illustrating the sensitivity analysis of the risk index of each Big Four snake species at the state level from present to different future climate scenarios. (TIF)

S28 Fig Area Under the Curve (AUC) values from the sensitivity analysis of the risk index for each of the Big Four snake species at the state level from present to projected future climate scenarios. (TIF)

S29 Fig Receiver Operating Characteristic (ROC) curve illustrating the sensitivity analysis of the risk index of each Big Four snake species at the district level from present to different future climate scenarios. (TIF)

S30 Fig Area Under the Curve (AUC) values from the sensitivity analysis of the risk index for each of the Big Four snake species at the district level from present to projected future climate scenarios. (TIF)

S1 Table This table lists all the initial variables considered in the study, along with their categories and data sources, prior to performing correlation analysis for variable selection. (XLSX)

S2 Table Model fit metrics for each of the participating modeling methods and for the final ensemble model for estimation of habitat suitability of the Big Four species. A total of five model algorithms were used with the threshold of < 0.75 AUC score. The models were Maximum Entropy (MaxEnt), Random Forest (RF), Boosted Regression Tree (BRT), Generalized Linear Model (GLM), and Multivariate Adaptive Regression Splines (MARS). AUC: Area under Curve, ΔAUC: Change in Area under curve (Training – Cross Validation), PCC: Proportion Correctly Classified, TSS: True Skill Statistic.
(XLSX)

S3 Table The mean percentage contribution of each covariate generated from the final ensemble model for the Big Four species. (XLSX)

S4 Table Habitat suitable extent (Class 5) of *B. caeruleus* in Indian states in Present and Future Climatic Scenarios (SSPs). (XLSX)

S5 Table Habitat suitable extent (Class 5) of *D. russelii* in Indian states in Present and Future Climatic Scenarios (SSPs). (XLSX)

S6 Table Habitat suitable extent (Class 5) of *E. carinatus* in Indian states in Present and Future Climatic Scenarios (SSPs). (XLSX)

S7 Table Habitat suitable extent (Class 5) of *N. naja* in Indian states in Present and Future Climatic Scenarios (SSPs). (XLSX)

S8 Table The area of cropland overlap (in sq. km. ) with the suitable habitat extent (Class 4 and Class 5 suitable extent) in present and future scenarios in Indian states for *B. caeruleus.*
(XLSX)

S9 Table The area of built-up overlap (in sq. km. ) with the suitable habitat extent (Class 4 and Class 5 suitable extent) in present and future scenarios in Indian states for *B. caeruleus.*
(XLSX)

S10 Table The area of cropland overlap (in sq. km. ) with the suitable habitat extent (Class 4 and Class 5 suitable extent) in present and future scenarios in Indian states for *D. russelii.*
(XLSX)

S11 Table The area of built-up overlap (in sq. km. ) with the suitable habitat extent (Class 4 and Class 5 suitable extent) in present and future scenarios in Indian states for *D. russelii.*
(XLSX)

S12 Table The area of cropland overlap (in sq. km. ) with the suitable habitat extent (Class 4 and Class 5 suitable extent) in present and future scenarios in Indian states for *E. carinatus*.
(XLSX)

S13 Table The area of built-up overlap (in sq. km. ) with the suitable habitat extent (Class 4 and Class 5 suitable extent) in present and future scenarios in Indian states for *E. carinatus.*
(XLSX)

S14 Table The area of cropland overlap (in sq. km. ) with the suitable habitat extent (Class 4 and Class 5 suitable extent) in present and future scenarios in Indian states for *N. naja.*
(XLSX)

S15 Table The area of built-up overlap (in sq. km. ) with the suitable habitat extent (Class 4 and Class 5 suitable extent) in present and future scenarios in Indian states for *N. naja*.
(XLSX)

S16 Table The risk index scores of each Indian state for *B. caeruleus* in present and future climatic scenarios. The high-risk index values underscore high priority for the state. The Growth Rate (GR) for each future scenario is calculated to determine the direction and magnitude of change relative to the present scenario.
(XLSX)

S17 Table The risk index scores of each Indian state for *D. russelii* in present and future climatic scenarios. The high-risk index values underscore high priority for the state. The Growth Rate (GR) for each future scenario is calculated to determine the direction and magnitude of change relative to the present scenario.
(XLSX)

S18 Table The risk index scores of each Indian state for *E. carinatus* in present and future climatic scenarios. The high-risk index values underscore high priority for the state. The Growth Rate (GR) for each future scenario is calculated to determine the direction and magnitude of change relative to the present scenario.
(XLSX)

S19 Table The risk index scores of each Indian state for *N. naja* in present and future climatic scenarios. The high-risk index values underscore high priority for the state. The Growth Rate (GR) for each future scenario is calculated to determine the direction and magnitude of change relative to the present scenario.
(XLSX)
