# Supplementary figures and images for: Future of snakebite risk in India: Consequence of climate change and the shifting habitats of the big four species in next five decades
Source: PLoS Negl Trop Dis. 2025 Sep 2;19(9):e0013464. doi: 10.1371/journal.pntd.0013464 (PMC12404559; doi:10.1371/journal.pntd.0013464)

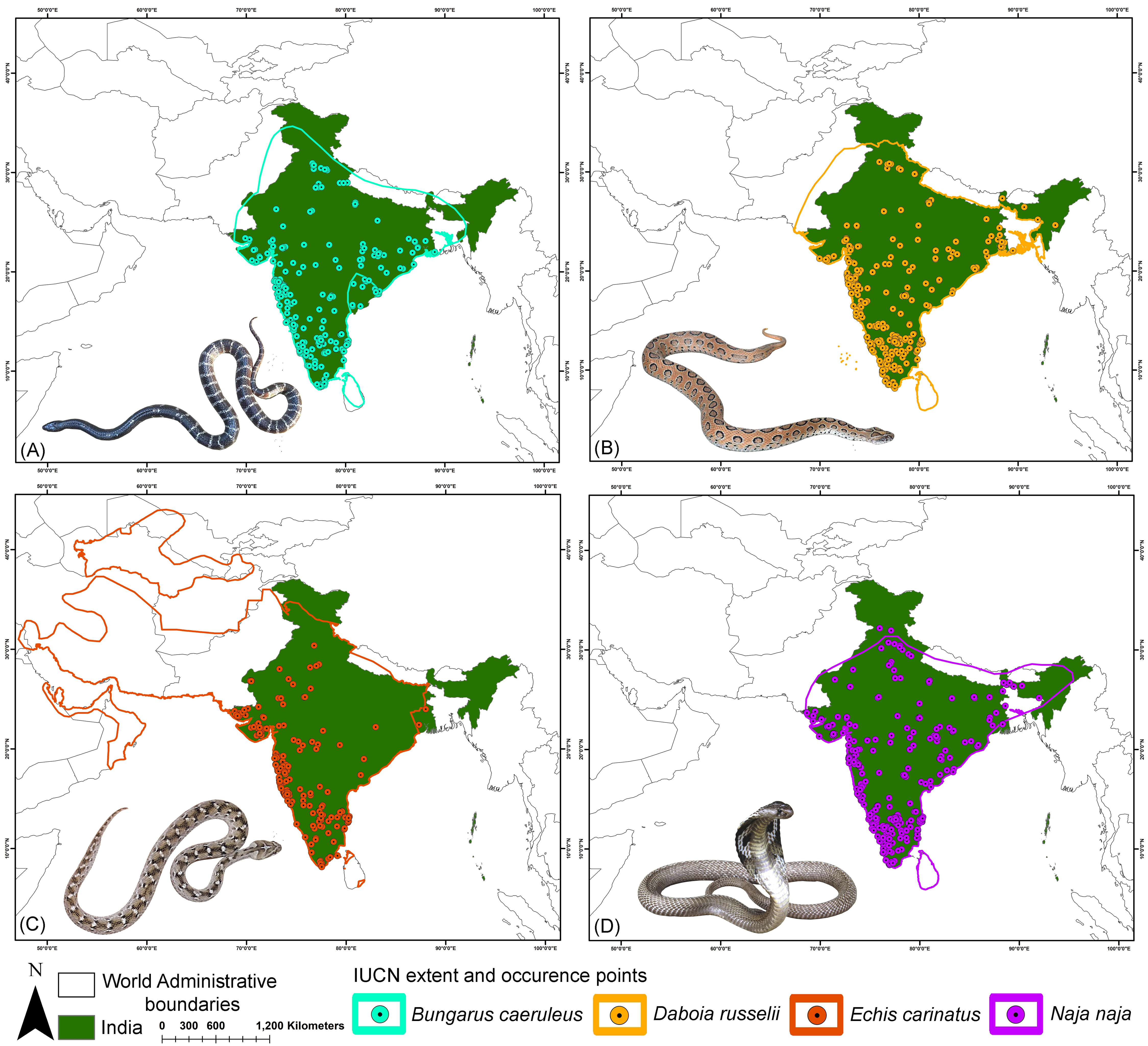

Supplement: S1 Fig — The administrative layer of the map was obtained from the DIVA-GIS website (https://diva-gis.org/data.html) and was created using ArcGIS software. The species photographs were provided by Anirban Chaudhuri and Raju Vyas through personal communication and were used with their prior permission. (TIF) [file pntd.0013464.s001.tif]

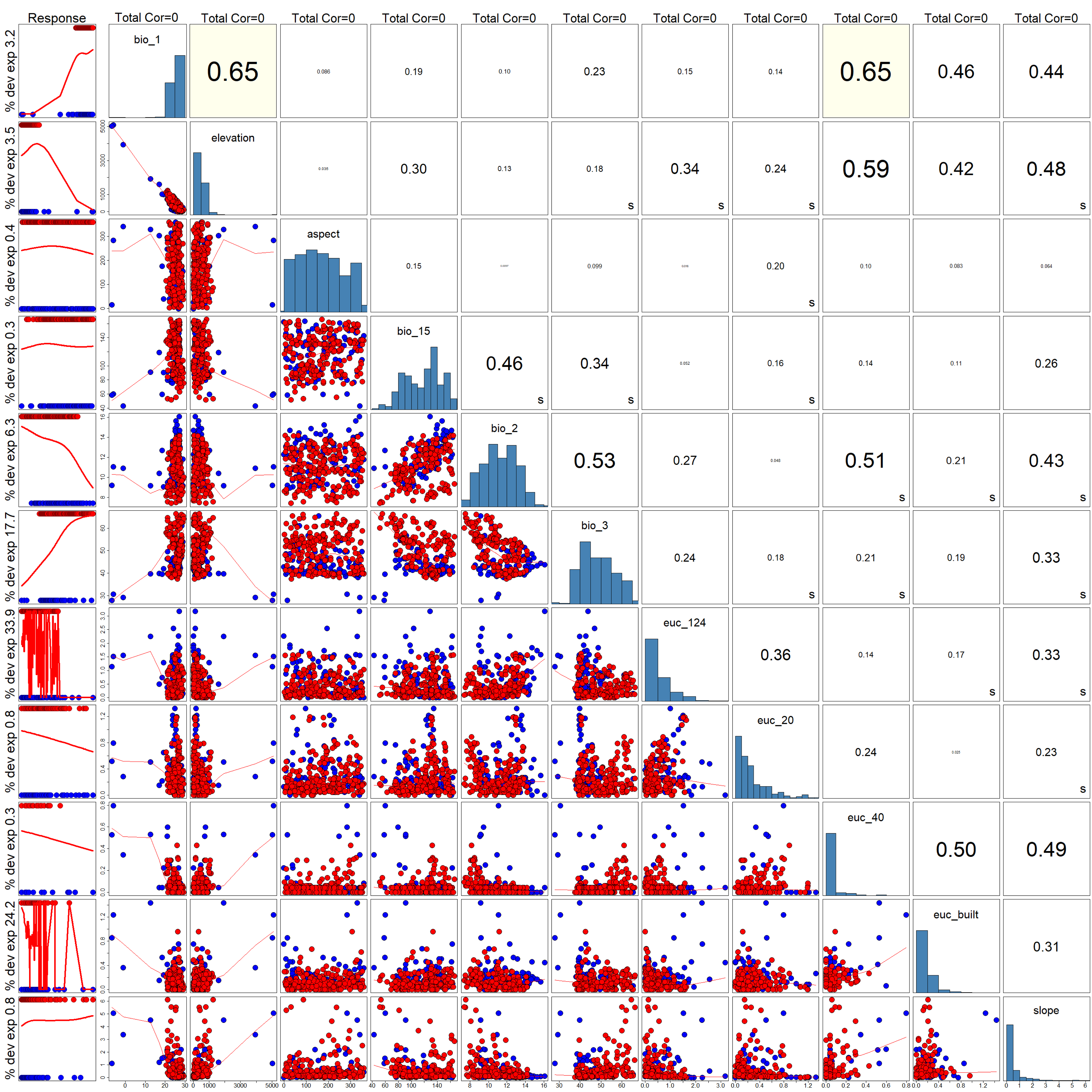

Supplement: S2 Fig — The figure illustrates the pairwise correlations (|r| < 0.8) among variables selected for B. caeruleus. The Pearson’s correlation coefficient is used as the primary measure. If either the Spearman or Kendall coefficient exceeds the Pearson value for a given pair, it is indicated with an “s” (Spearman) or “k” (Kendall) in the bottom-right corner of the corresponding cell. (TIF) [file pntd.0013464.s002.tif]

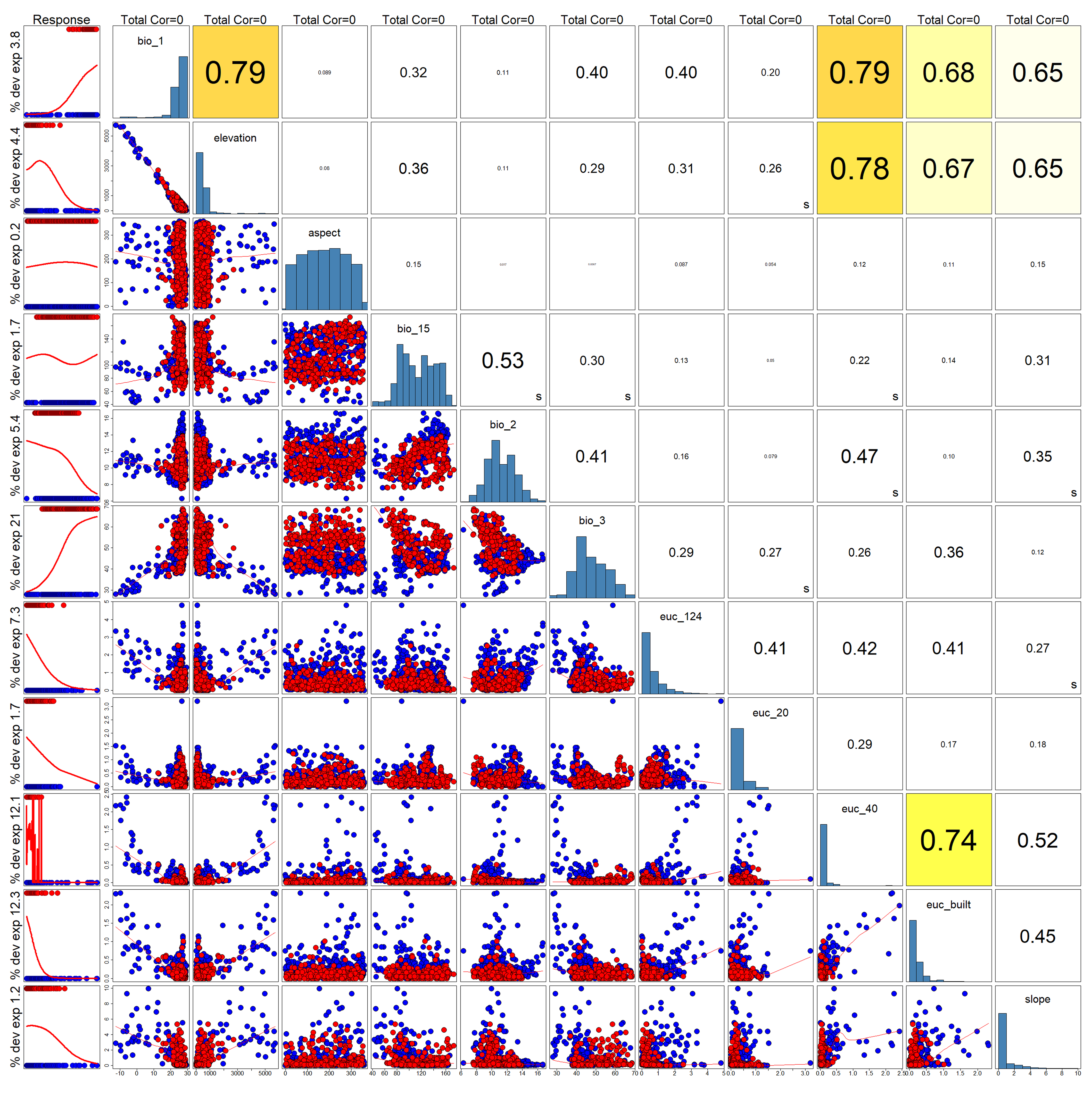

Supplement: S3 Fig — The figure illustrates the pairwise correlations (|r| < 0.8) among variables selected for D. russelii. The Pearson’s correlation coefficient is used as the primary measure. If either the Spearman or Kendall coefficient exceeds the Pearson value for a given pair, it is indicated with an “s” (Spearman) or “k” (Kendall) in the bottom-right corner of the corresponding cell. (TIF) [file pntd.0013464.s003.tif]

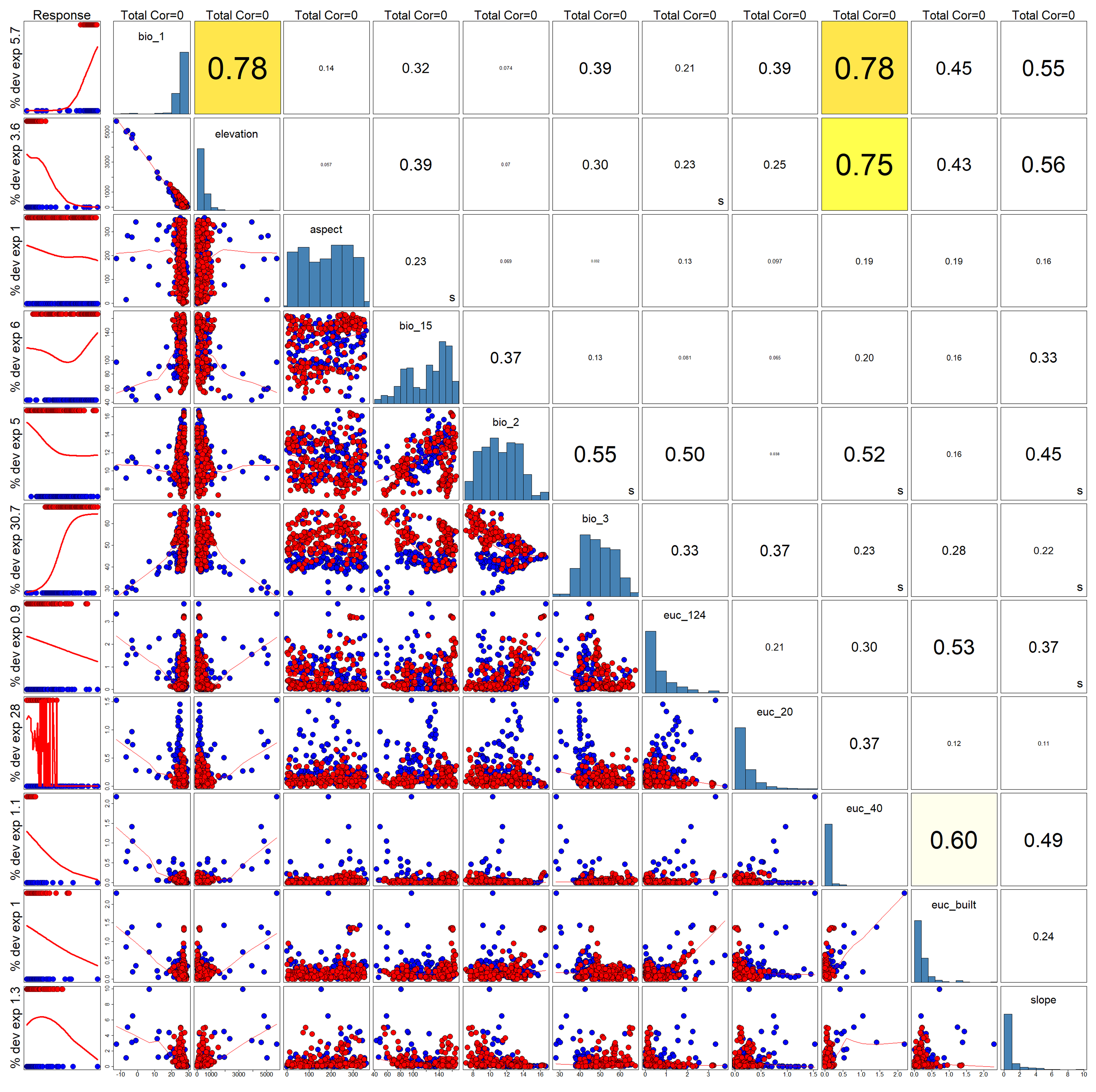

Supplement: S4 Fig — The figure illustrates the pairwise correlations (|r| < 0.8) among variables selected for E. carinatus. The Pearson’s correlation coefficient is used as the primary measure. If either the Spearman or Kendall coefficient exceeds the Pearson value for a given pair, it is indicated with an “s” (Spearman) or “k” (Kendall) in the bottom-right corner of the corresponding cell. (TIF) [file pntd.0013464.s004.tif]

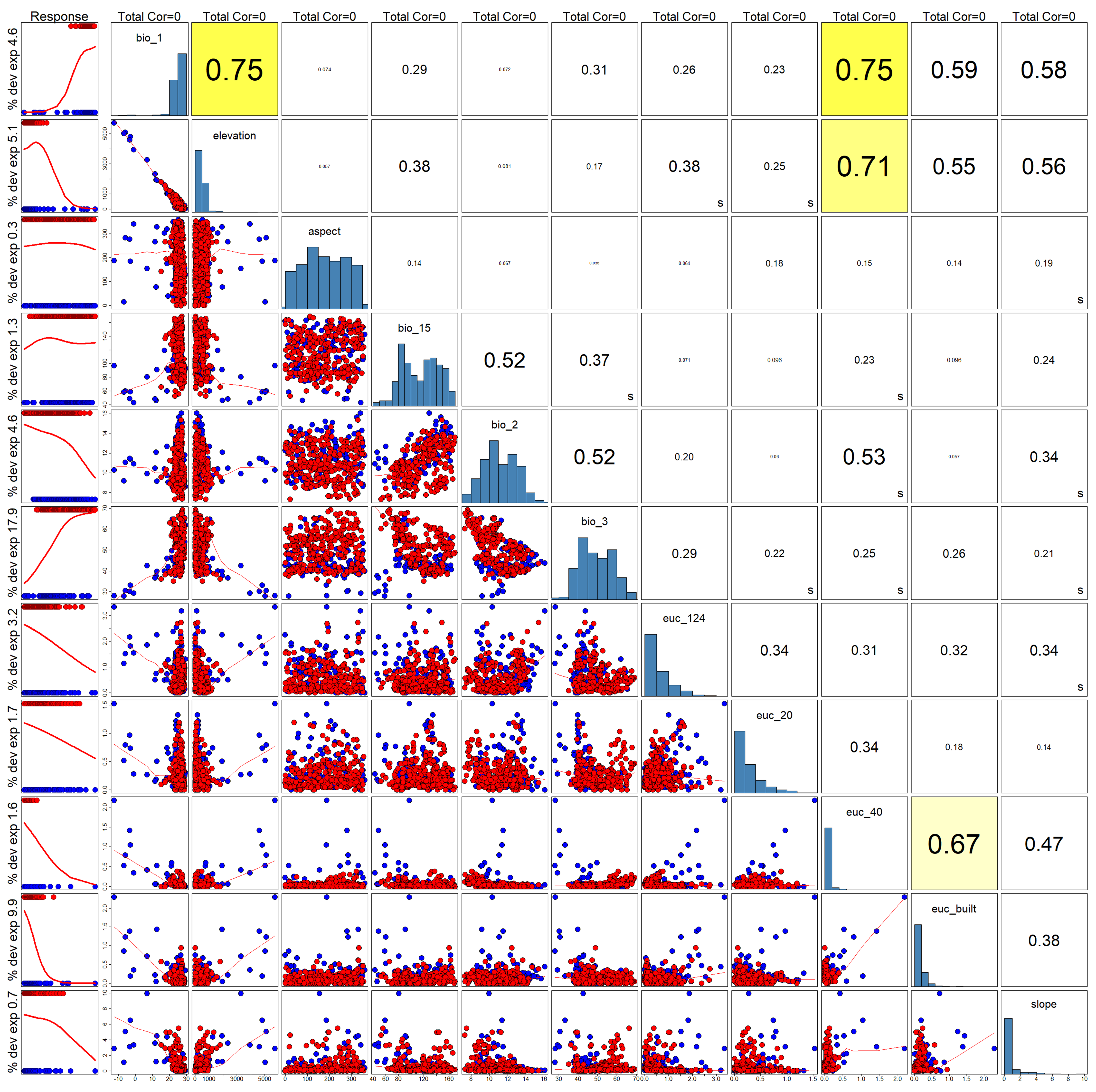

Supplement: S5 Fig — The figure illustrates the pairwise correlations (|r| < 0.8) among variables selected for N. naja. The Pearson’s correlation coefficient is used as the primary measure. If either the Spearman or Kendall coefficient exceeds the Pearson value for a given pair, it is indicated with an “s” (Spearman) or “k” (Kendall) in the bottom-right corner of the corresponding cell. (TIF) [file pntd.0013464.s005.tif]

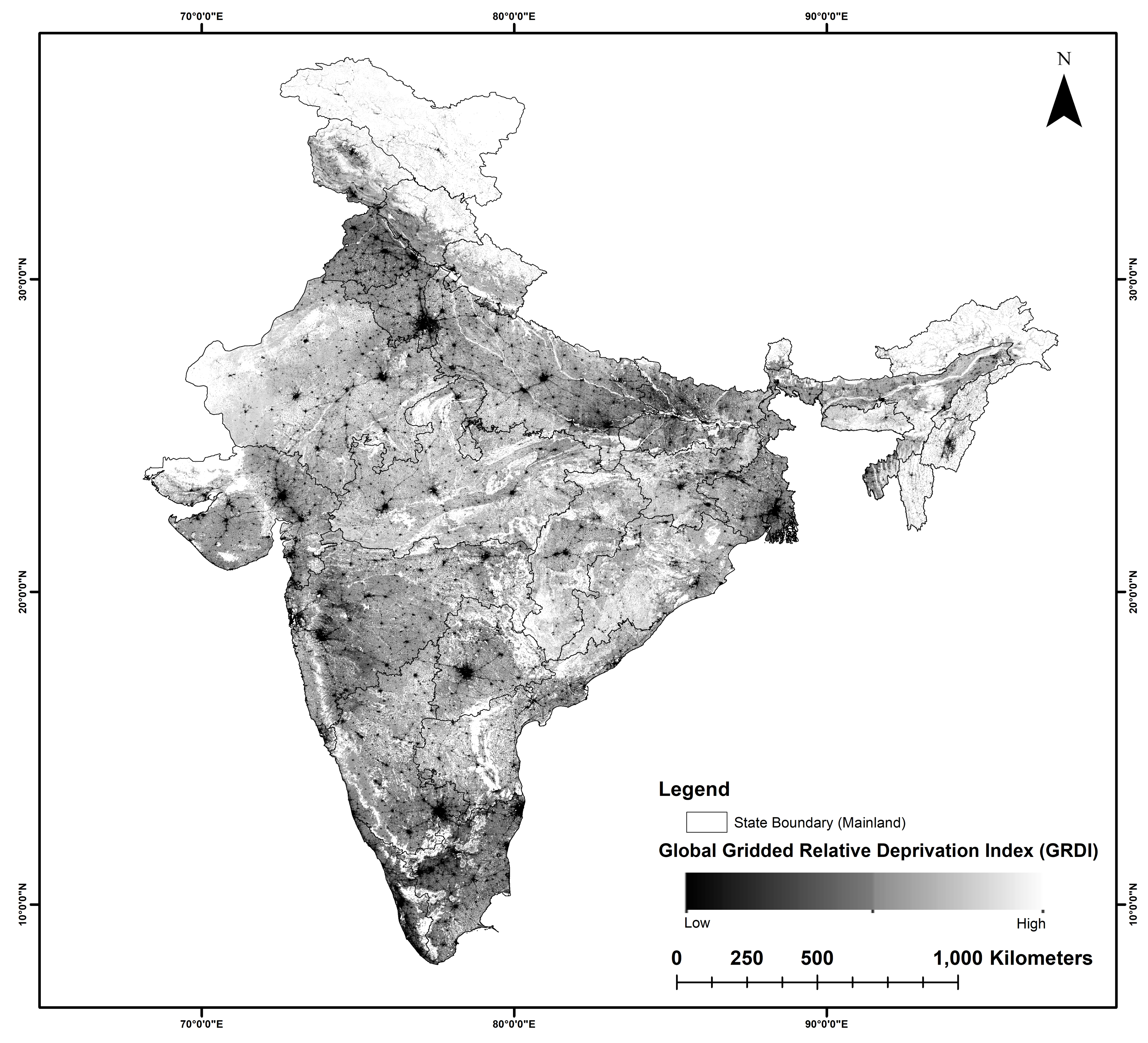

Supplement: S6 Fig — The figure determines the Global Gridded Relative Deprivation Index (GRDI) v1 (2010–2020) obtained from NASA’s Socioeconomic Data and Applications Center (SEDAC) (https://cmr.earthdata.nasa.gov/). The administrative layer of the map was obtained from the DIVA-GIS website (https://diva-gis.org/data.html) and was created using ArcGIS software. (TIF) [file pntd.0013464.s006.tif]

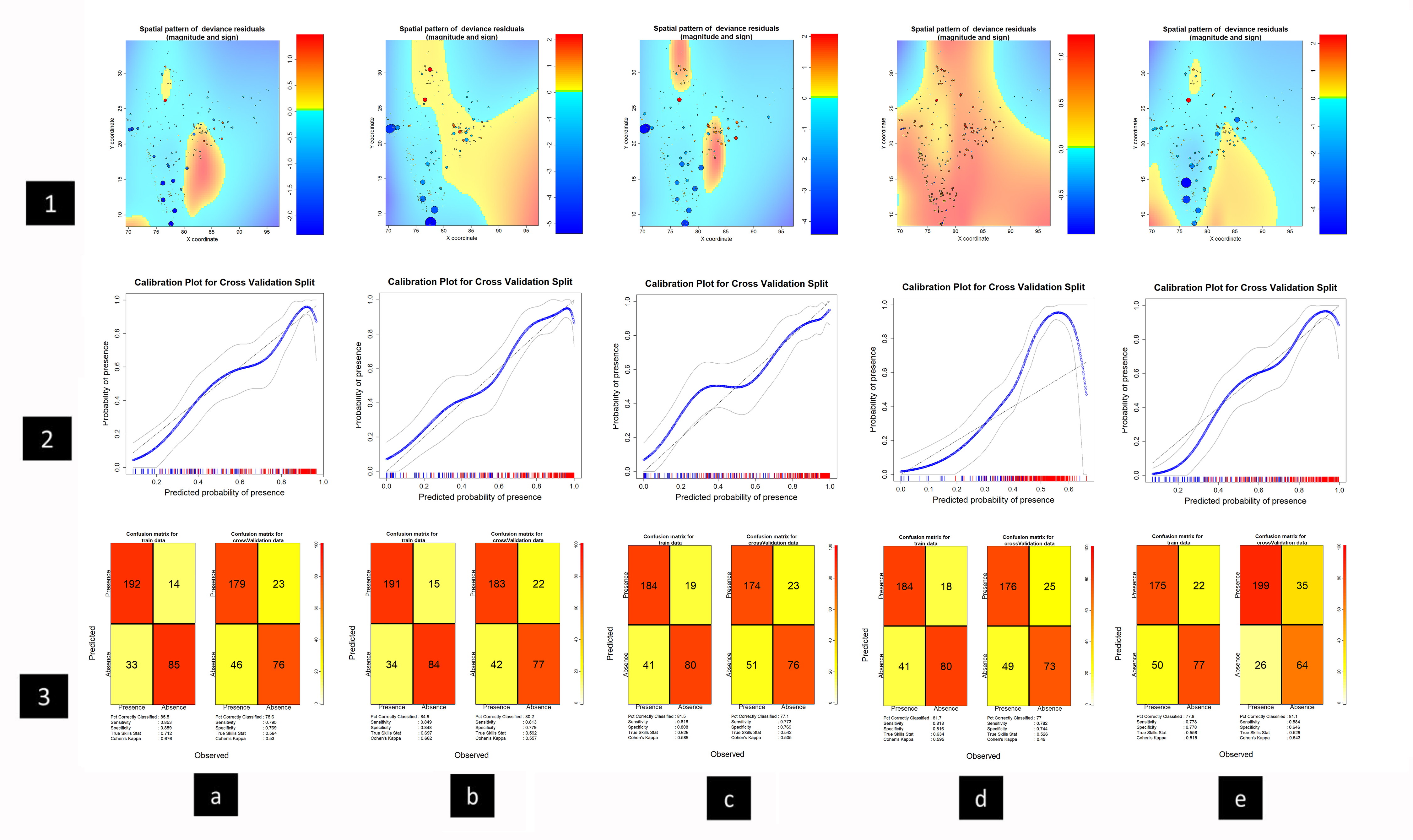

Supplement: S7 Fig — Row 1 represents spatial pattern of residuals where colour ramp indicates the magnitude of deviance and size represents the quantity. Row 2 represents model calibration plot across all 5 different model for cross-validation split. Row 3 represents confusion matrix for all 5 models, plotted by observed vs. predicted where colour ramp from lowest value 0% (white) to 100% (red) indicates the quantification of particular pair types. Column a. represents plots for BRT, Column b. represents plots for GLM, Column c. represents plots for MARS, Column d. represents plots for MaxEnt and Column e. represents plots for RF. (TIF) [file pntd.0013464.s007.tif]

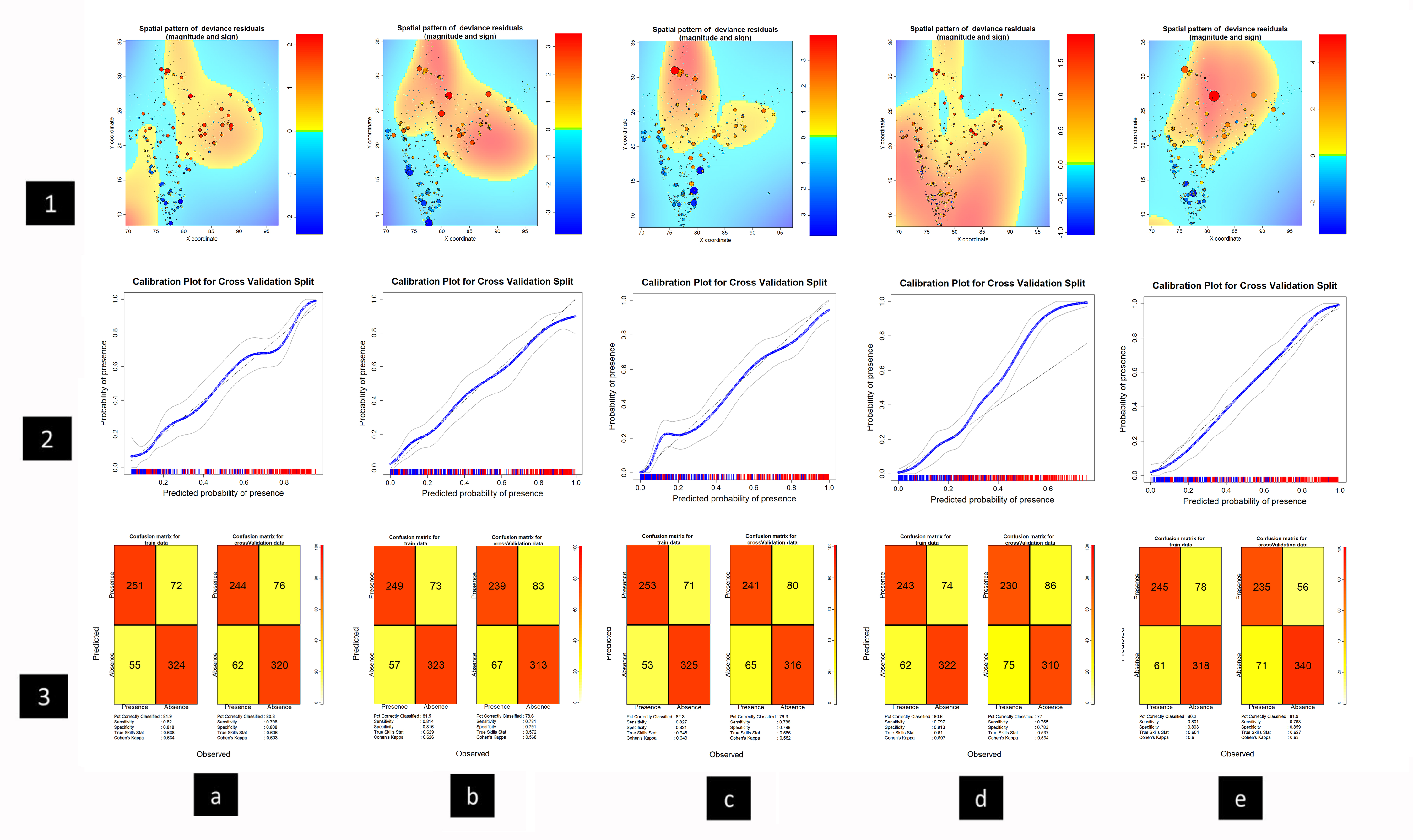

Supplement: S8 Fig — Row 1 represents spatial pattern of residuals where colour ramp indicates the magnitude of deviance and size represents the quantity. Row 2 represents model calibration plot across all 5 different model for cross-validation split. Row 3 represents confusion matrix for all 5 models, plotted by observed vs. predicted where colour ramp from lowest value 0% (white) to 100% (red) indicates the quantification of particular pair types. Column a. represents plots for BRT, Column b. represents plots for GLM, Column c. represents plots for MARS, Column d. represents plots for MaxEnt and Column e. represents plots for RF. (TIF) [file pntd.0013464.s008.tif]

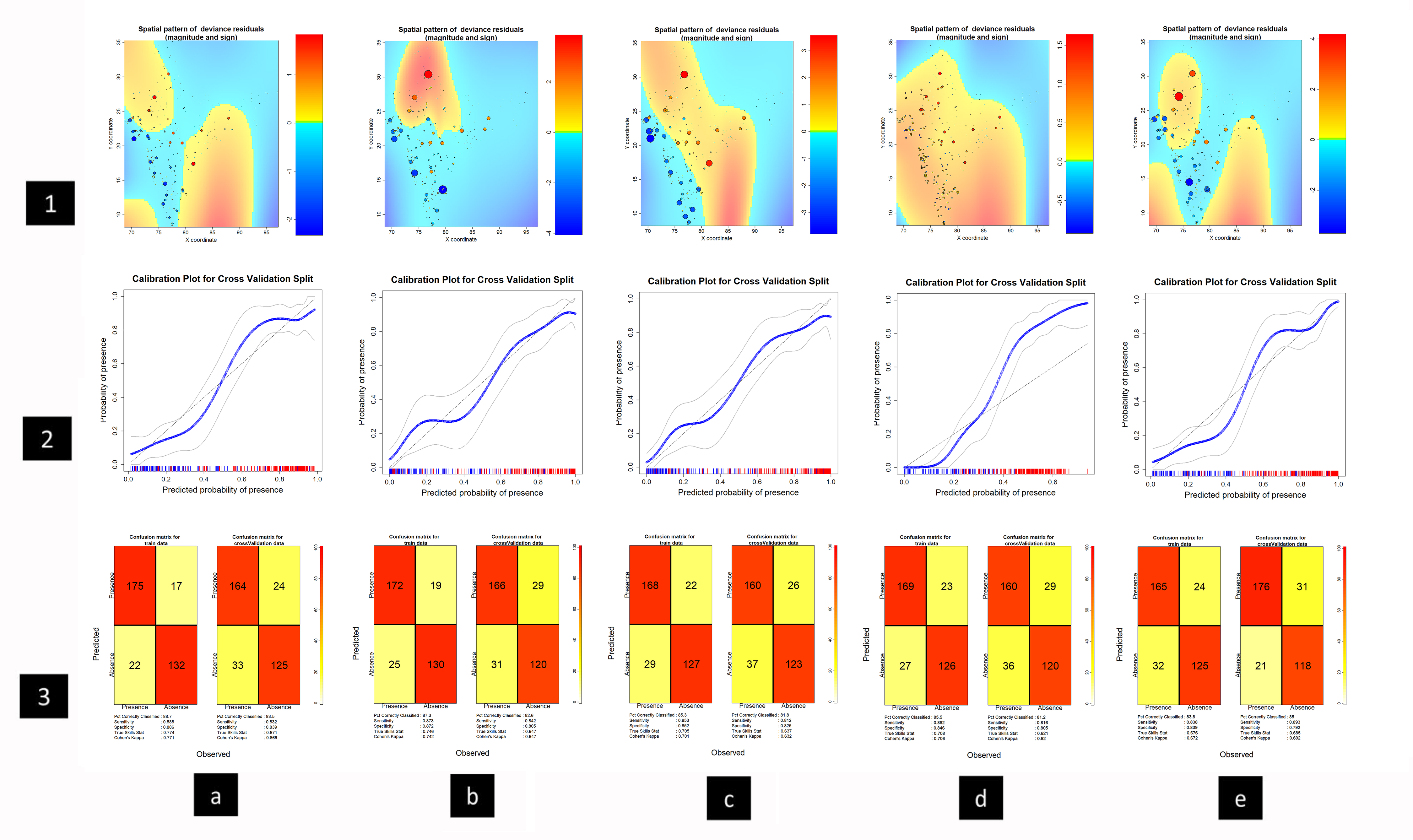

Supplement: S9 Fig — Row 1 represents spatial pattern of residuals where colour ramp indicates the magnitude of deviance and size represents the quantity. Row 2 represents model calibration plot across all 5 different model for cross-validation split. Row 3 represents confusion matrix for all 5 models, plotted by observed vs. predicted where colour ramp from lowest value 0% (white) to 100% (red) indicates the quantification of particular pair types. Column a. represents plots for BRT, Column b. represents plots for GLM, Column c. represents plots for MARS, Column d. represents plots for MaxEnt and Column e. represents plots for RF. (TIF) [file pntd.0013464.s009.tif]

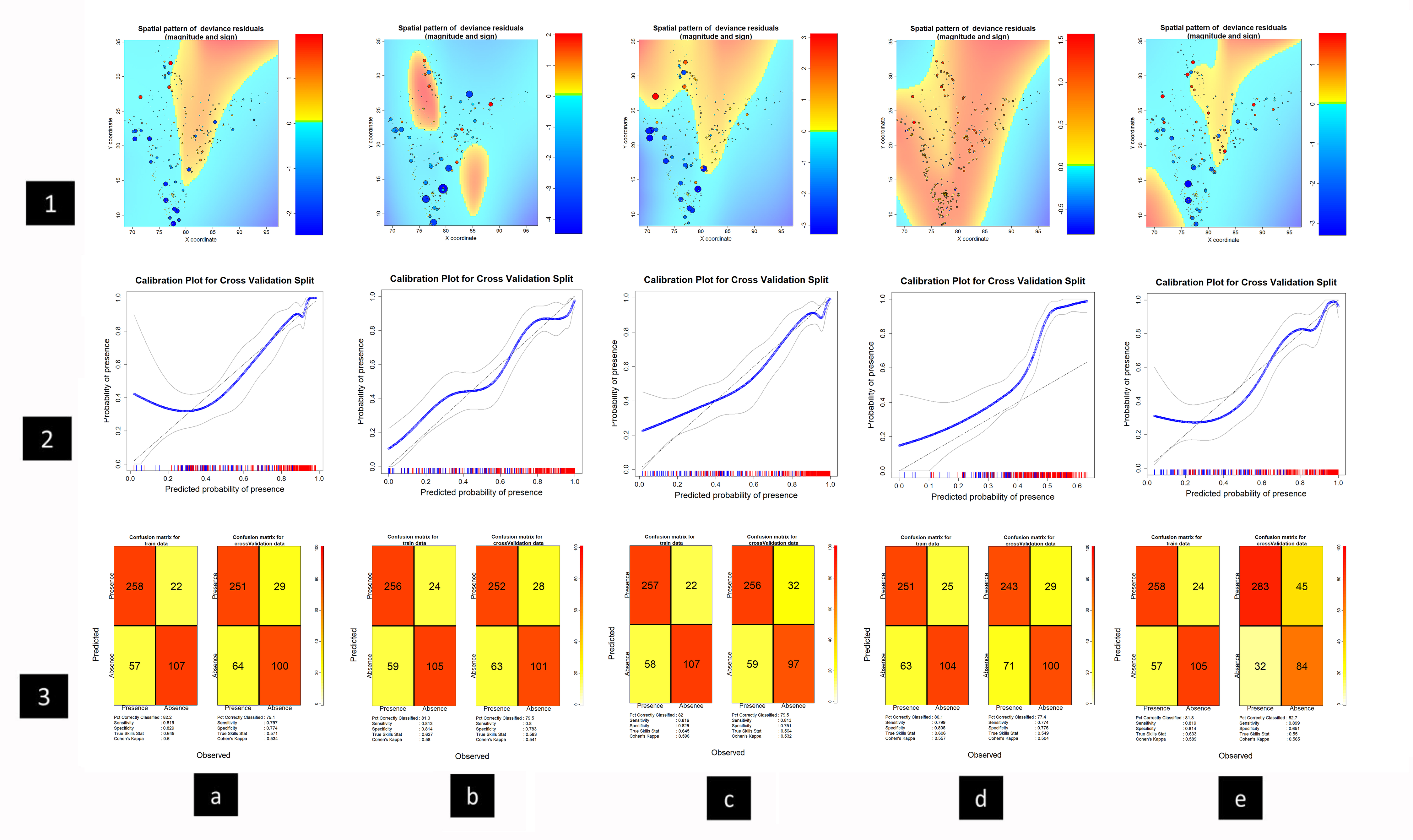

Supplement: S10 Fig — Row 1 represents spatial pattern of residuals where colour ramp indicates the magnitude of deviance and size represents the quantity. Row 2 represents model calibration plot across all 5 different model for cross-validation split. Row 3 represents confusion matrix for all 5 models, plotted by observed vs. predicted where colour ramp from lowest value 0% (white) to 100% (red) indicates the quantification of particular pair types. Column a. represents plots for BRT, Column b. represents plots for GLM, Column c. represents plots for MARS, Column d. represents plots for MaxEnt and Column e. represents plots for RF. (TIF) [file pntd.0013464.s010.tif]

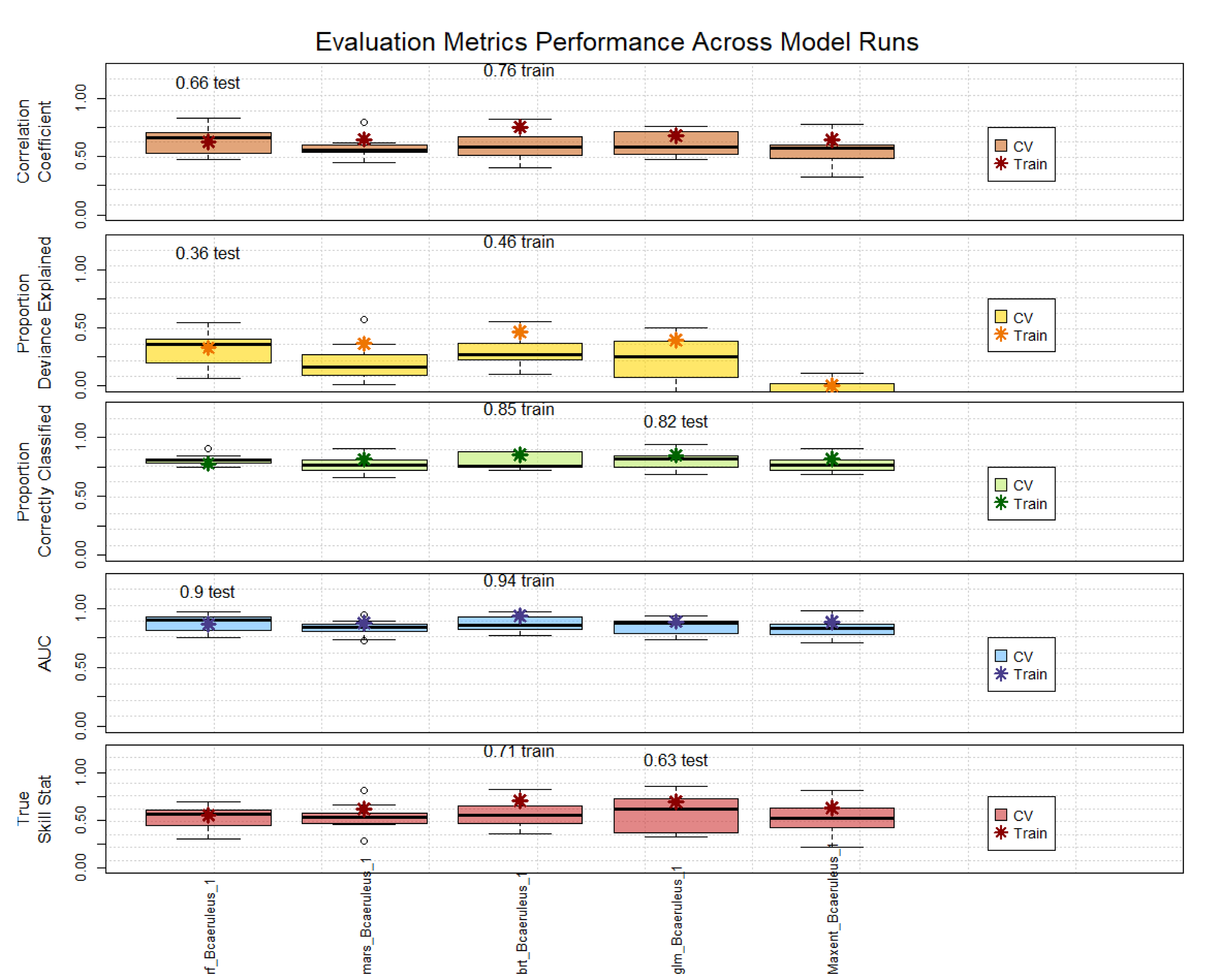

Supplement: S11 Fig — Brown - represents the correlation coefficient among the 5 different models. Yellow - represents the proportion of deviance explained; Green - represents the Proportion of correctly classified; Blue - represents Area under curve (AUC) and Pink - represents true skill statistics. (TIF) [file pntd.0013464.s011.tif]

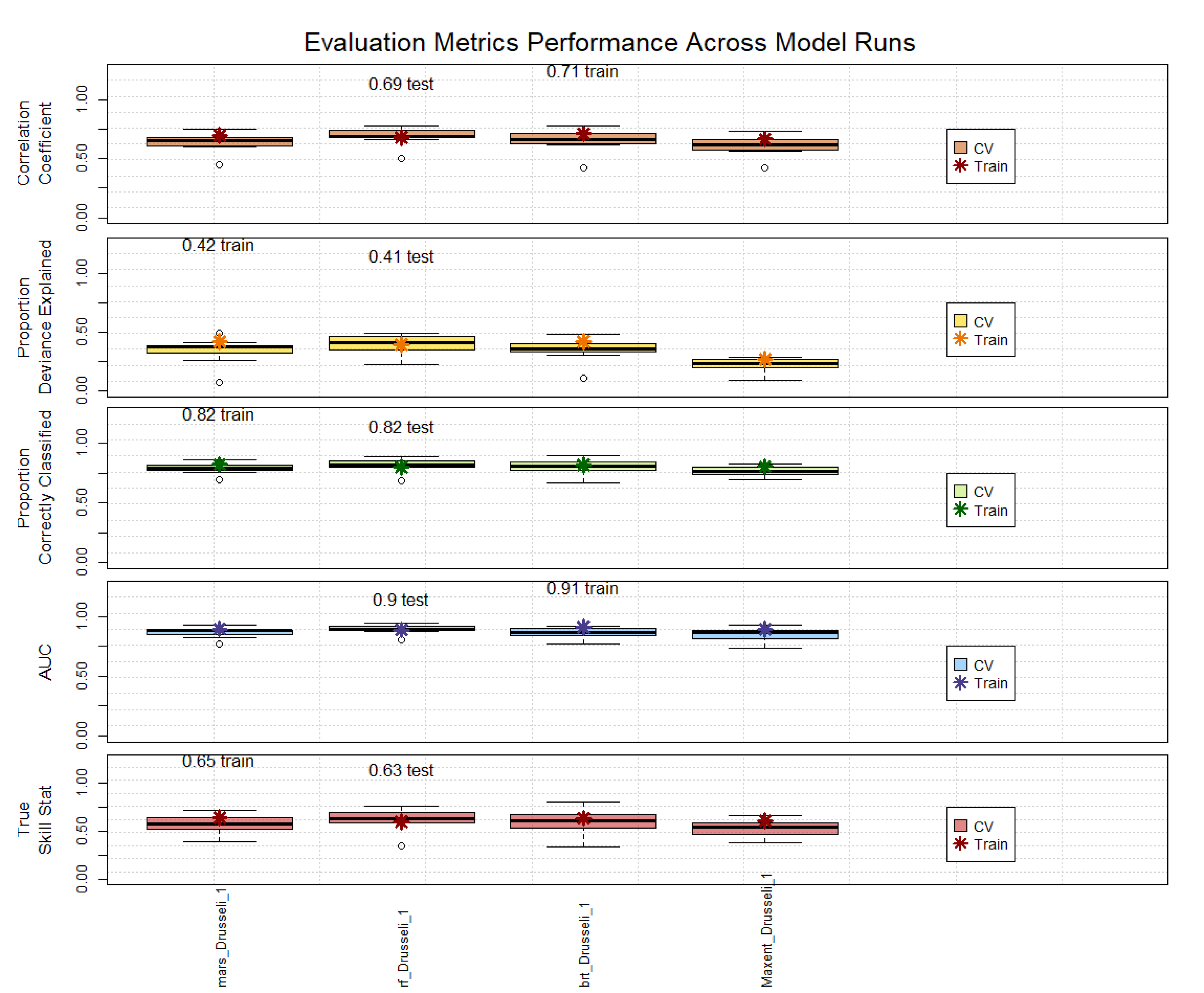

Supplement: S12 Fig — Brown - represents the correlation coefficient among the 5 different models. Yellow - represents the proportion of deviance explained; Green - represents the Proportion of correctly classified; Blue - represents Area under curve (AUC) and Pink - represents true skill statistics. (TIF) [file pntd.0013464.s012.tif]

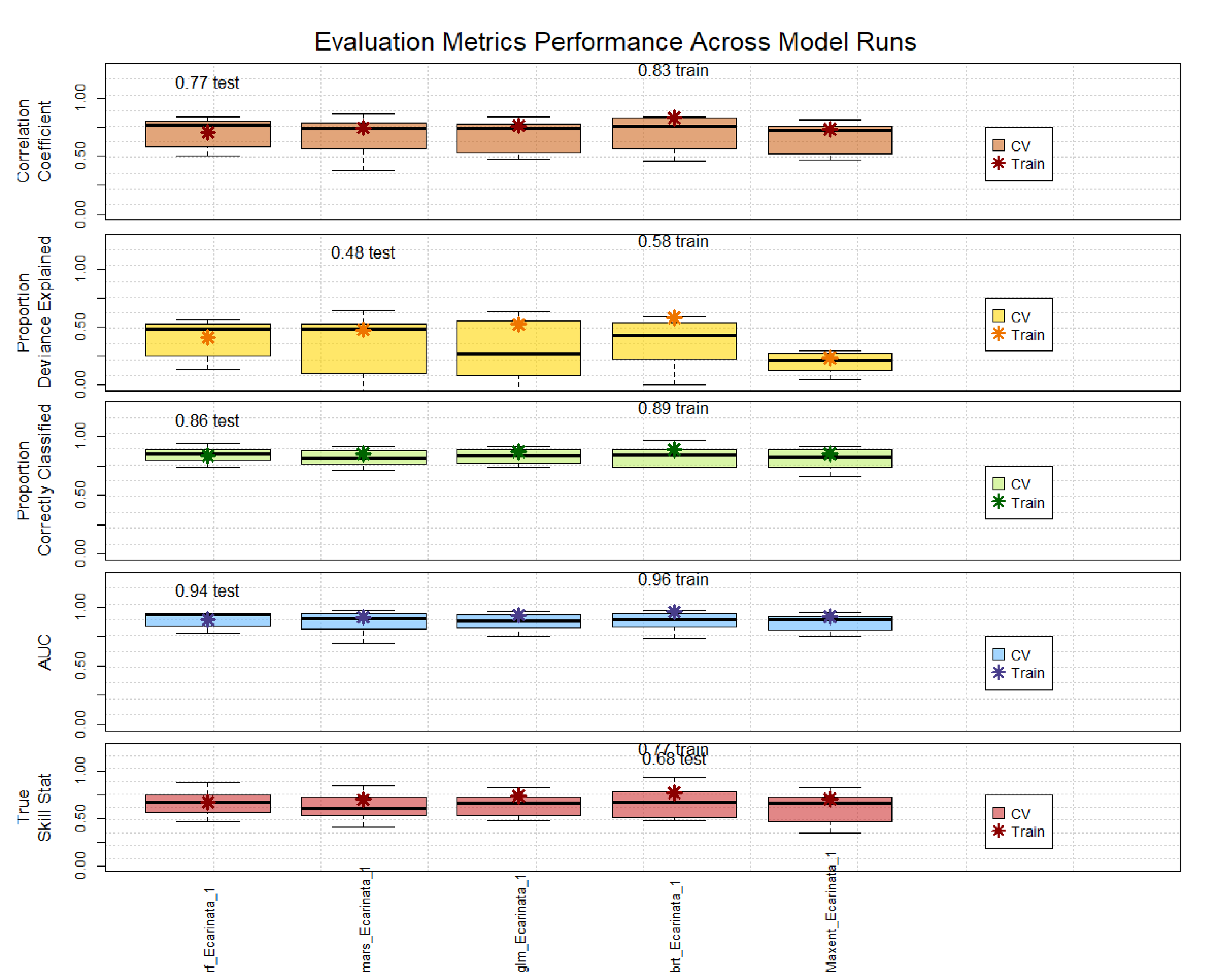

Supplement: S13 Fig — Brown - represents the correlation coefficient among the 5 different models. Yellow - represents the proportion of deviance explained; Green - represents the Proportion of correctly classified; Blue - represents Area under curve (AUC) and Pink - represents true skill statistics. (TIF) [file pntd.0013464.s013.tif]

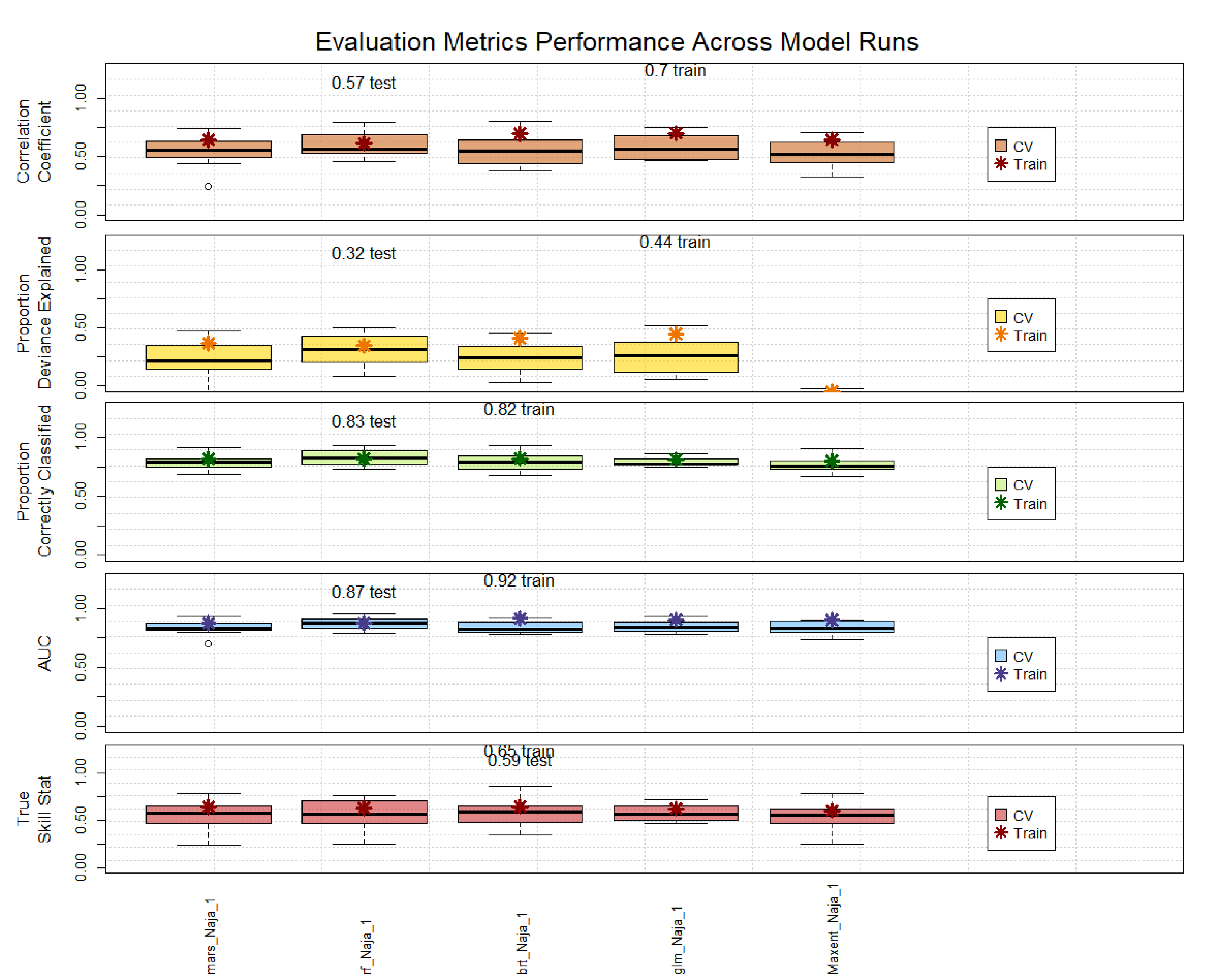

Supplement: S14 Fig — Brown - represents the correlation coefficient among the 5 different models. Yellow - represents the proportion of deviance explained; Green - represents the Proportion of correctly classified; Blue - represents Area under curve (AUC) and Pink - represents true skill statistics. (TIF) [file pntd.0013464.s014.tif]

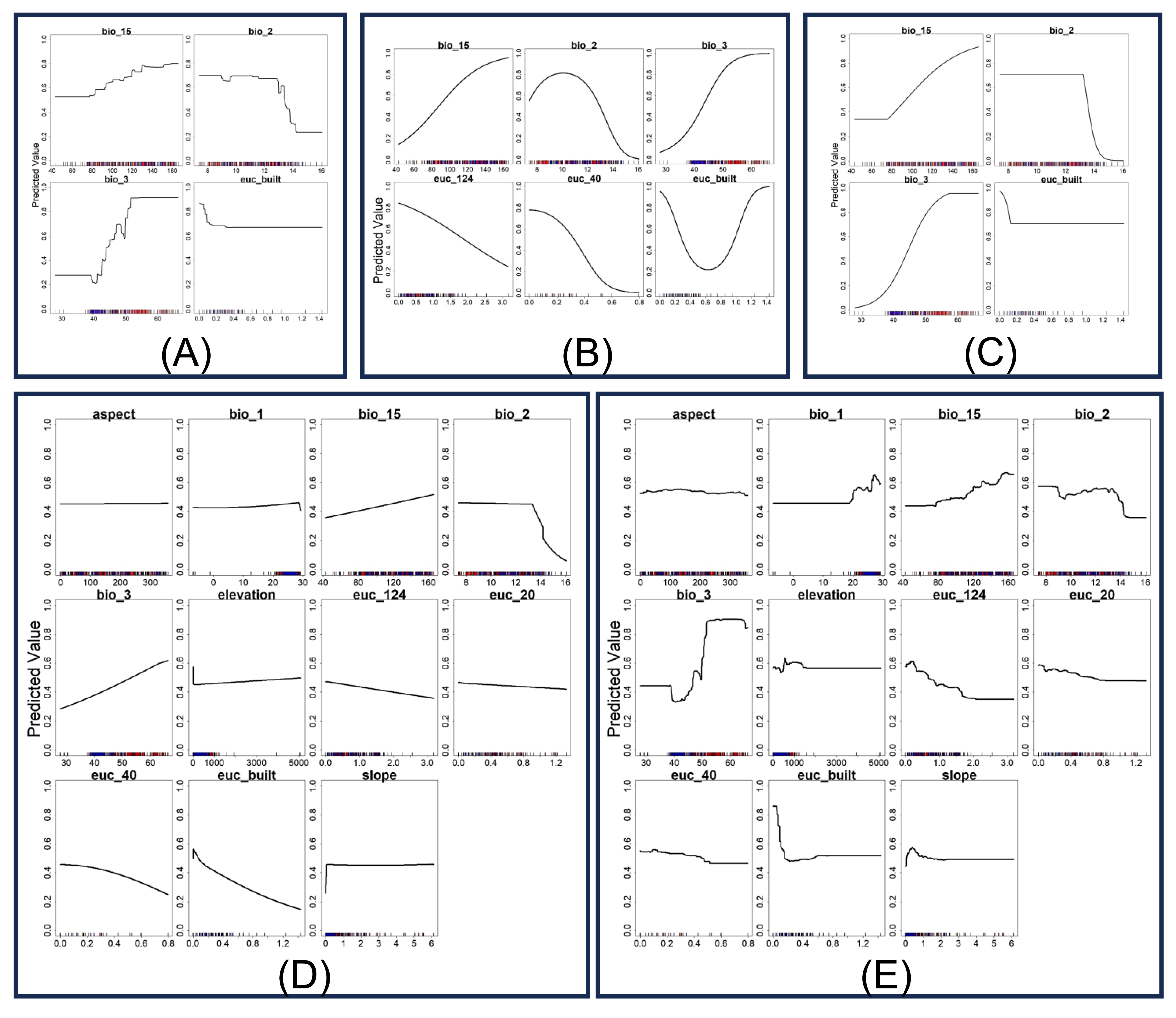

Supplement: S15 Fig — (A) BRT, (B) GLM, (C) MARS, (D) MAXENT, and (E) RF. (TIF) [file pntd.0013464.s015.tif]

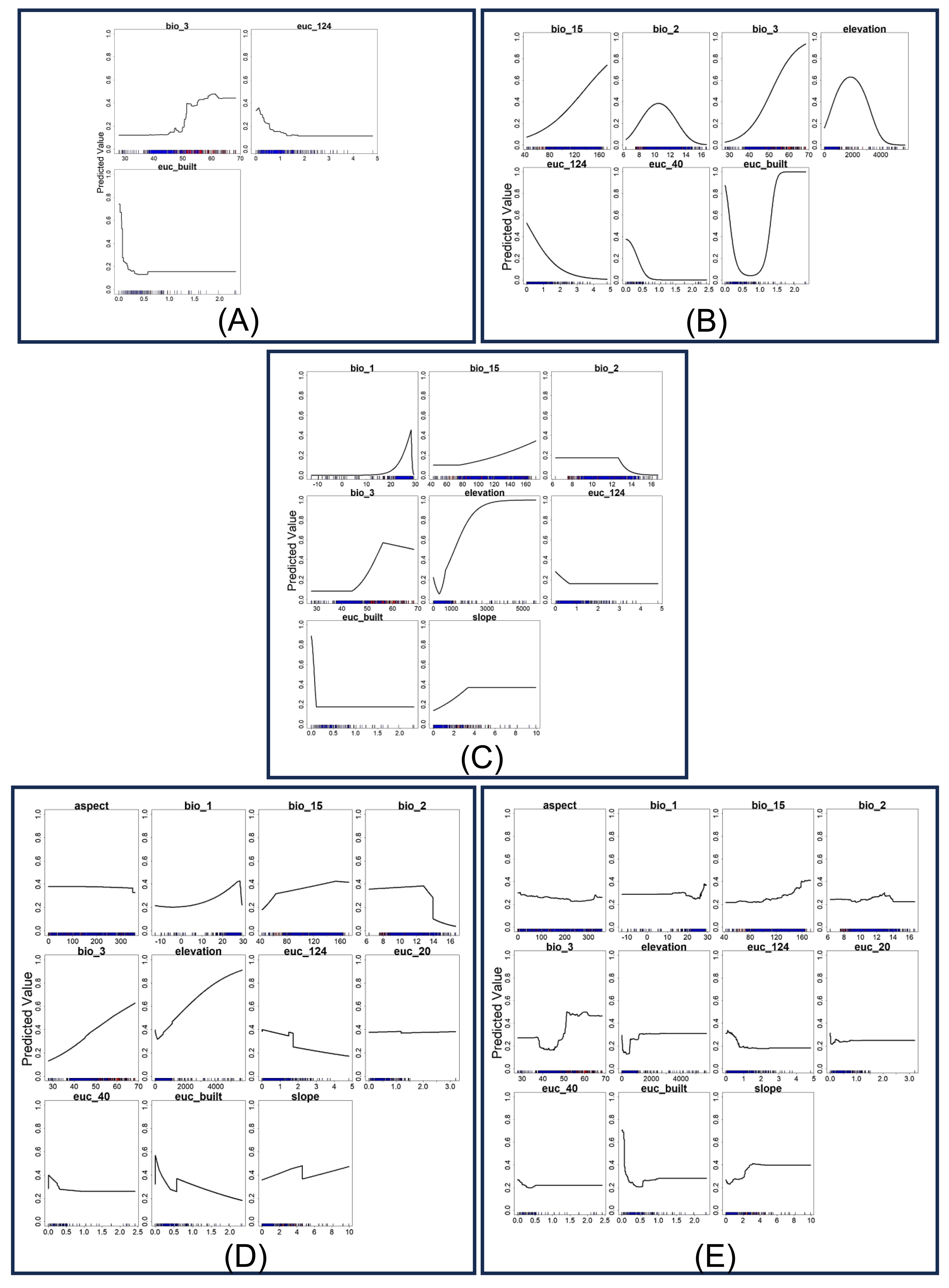

Supplement: S16 Fig — (A) BRT, (B) GLM, (C) MARS, (D) MAXENT, and (E) RF. (TIF) [file pntd.0013464.s016.tif]

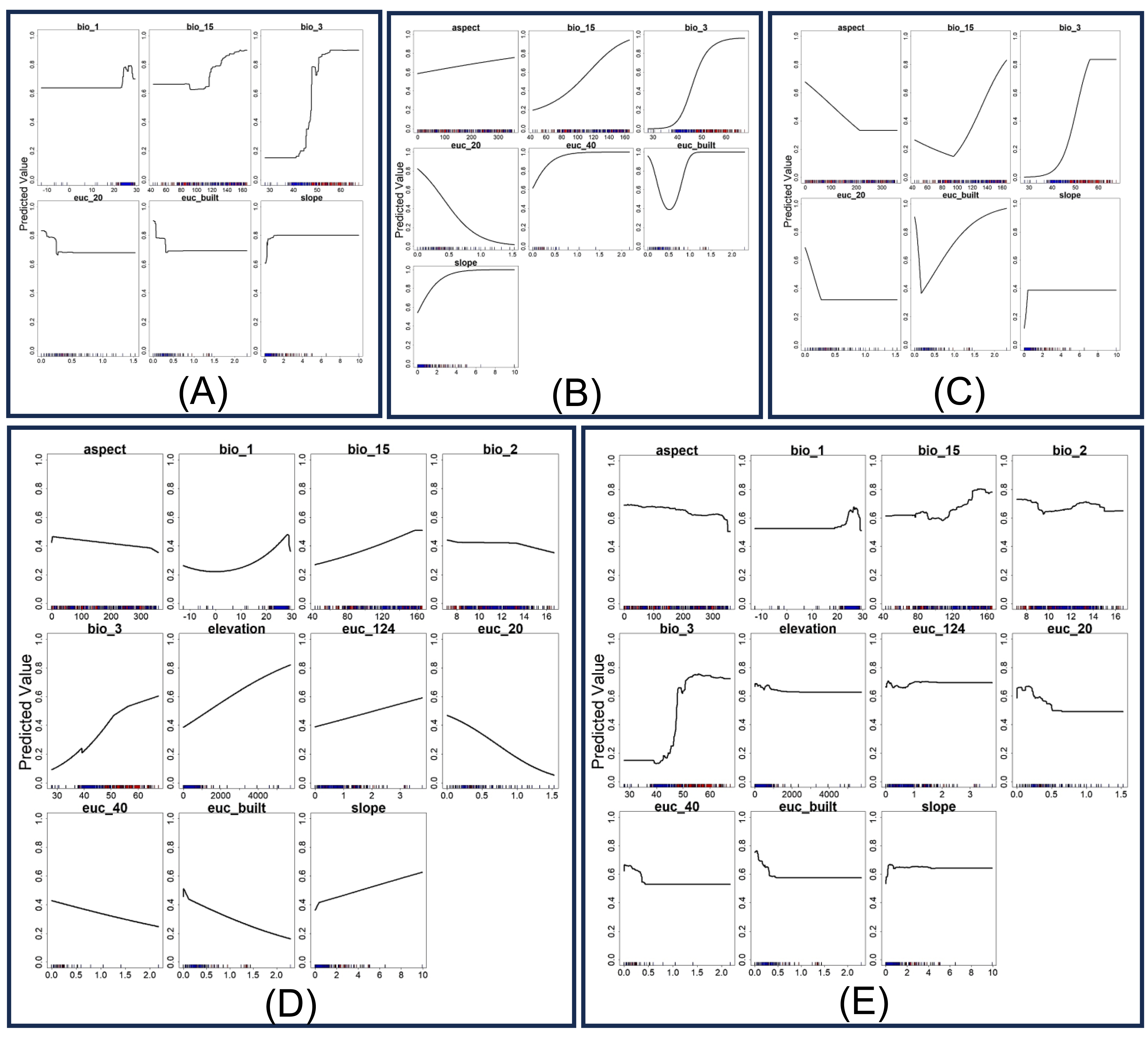

Supplement: S17 Fig — (A) BRT, (B) GLM, (C) MARS, (D) MAXENT, and (E) RF. (TIF) [file pntd.0013464.s017.tif]

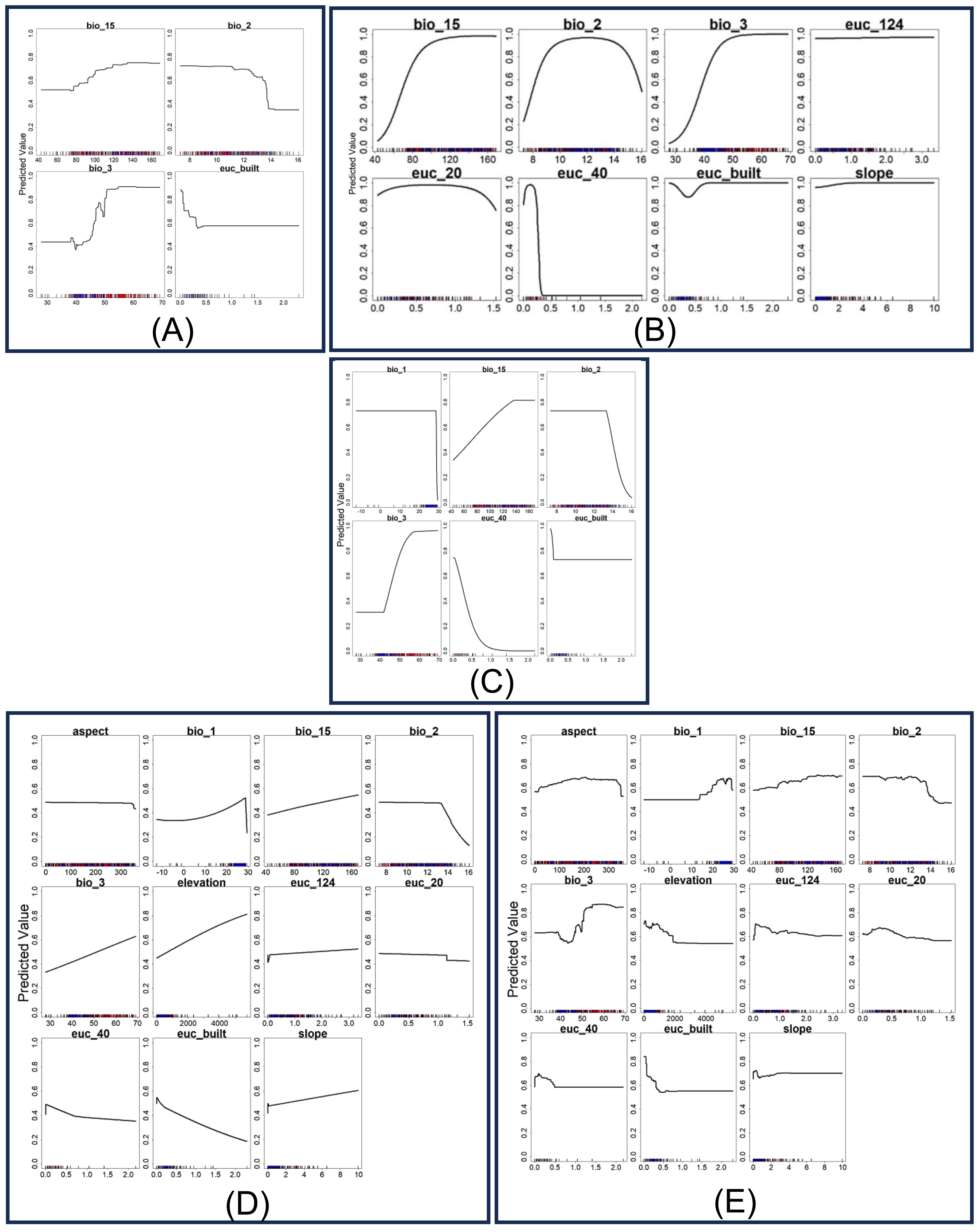

Supplement: S18 Fig — (A) BRT, (B) GLM, (C) MARS, (D) MAXENT, and (E) RF. (TIF) [file pntd.0013464.s018.tif]

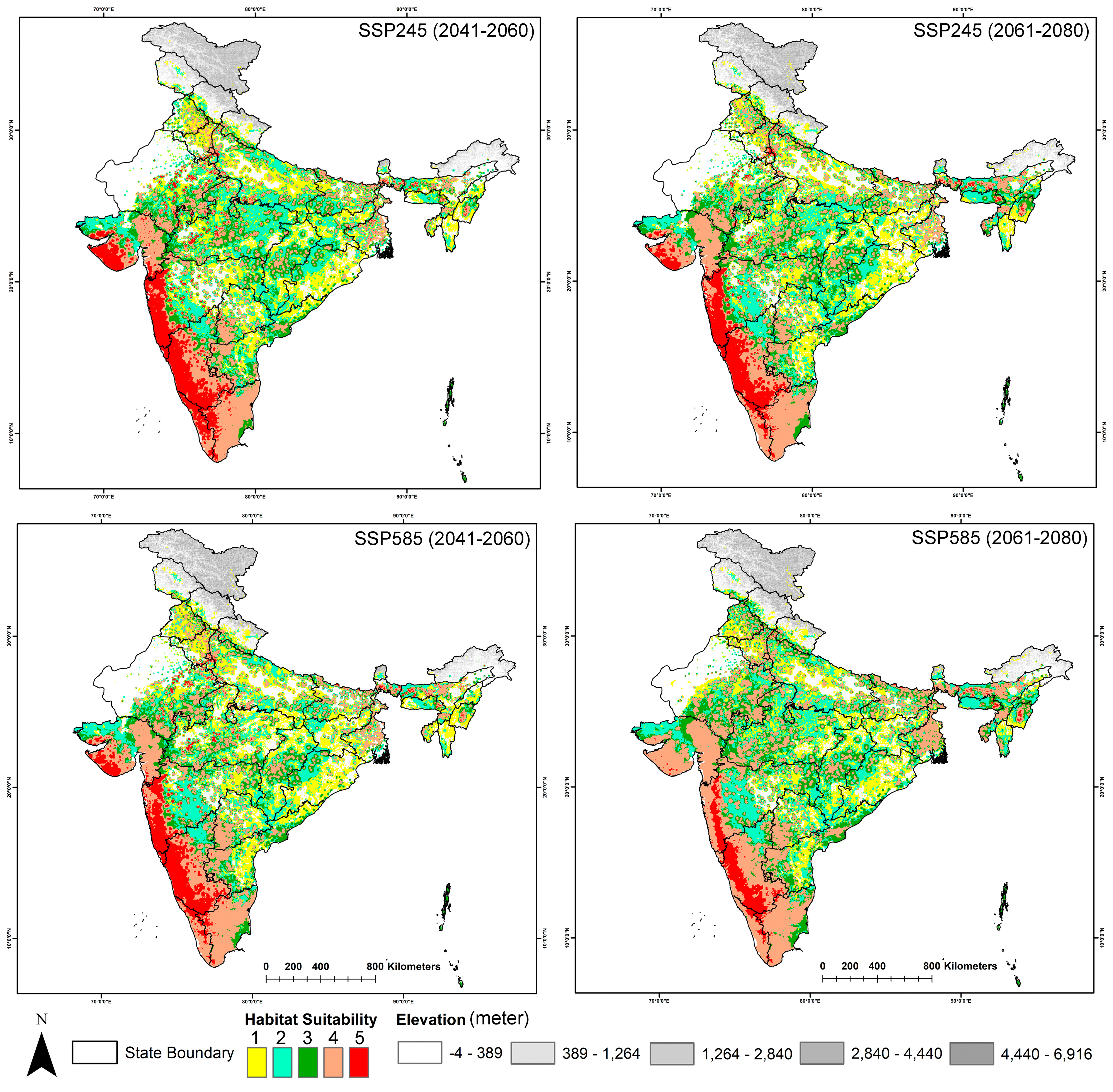

Supplement: S19 Fig — Here the ‘class 5’ determines the extremely suitable habitat extent. The administrative layer of the map was obtained from the DIVA-GIS website (https://diva-gis.org/data.html). The base layer of the maps is an elevation raster sourced from the SRTM website (http://srtm.csi.cgiar.org/srtmdata/) and was created using ArcGIS software. (TIF) [file pntd.0013464.s019.tif]

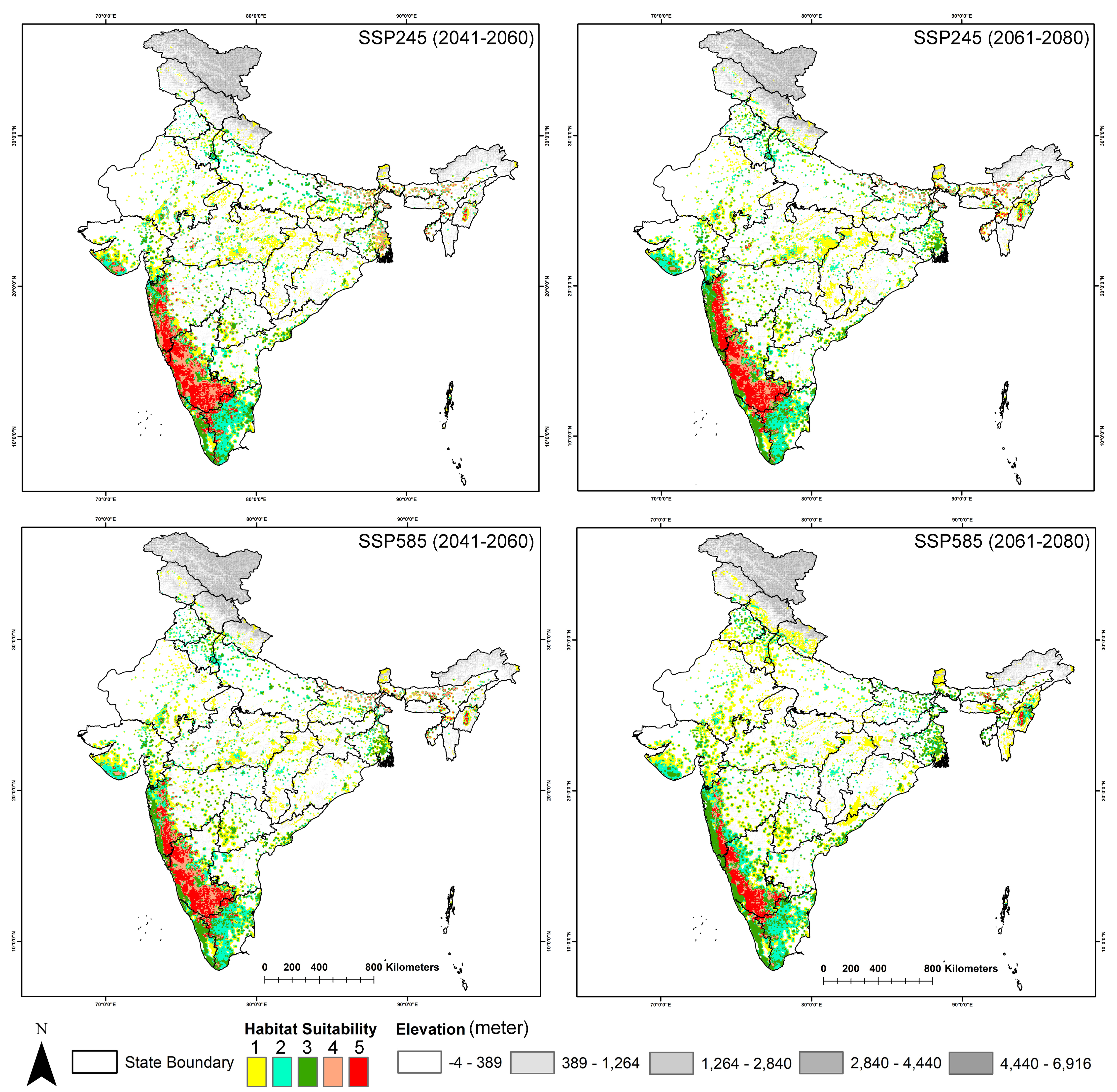

Supplement: S20 Fig — Here the ‘class 5’ determines the extremely suitable habitat extent. The administrative layer of the map was obtained from the DIVA-GIS website (https://diva-gis.org/data.html). The base layer of the maps is an elevation raster sourced from the SRTM website (http://srtm.csi.cgiar.org/srtmdata/) and was created using ArcGIS software. (TIF) [file pntd.0013464.s020.tif]

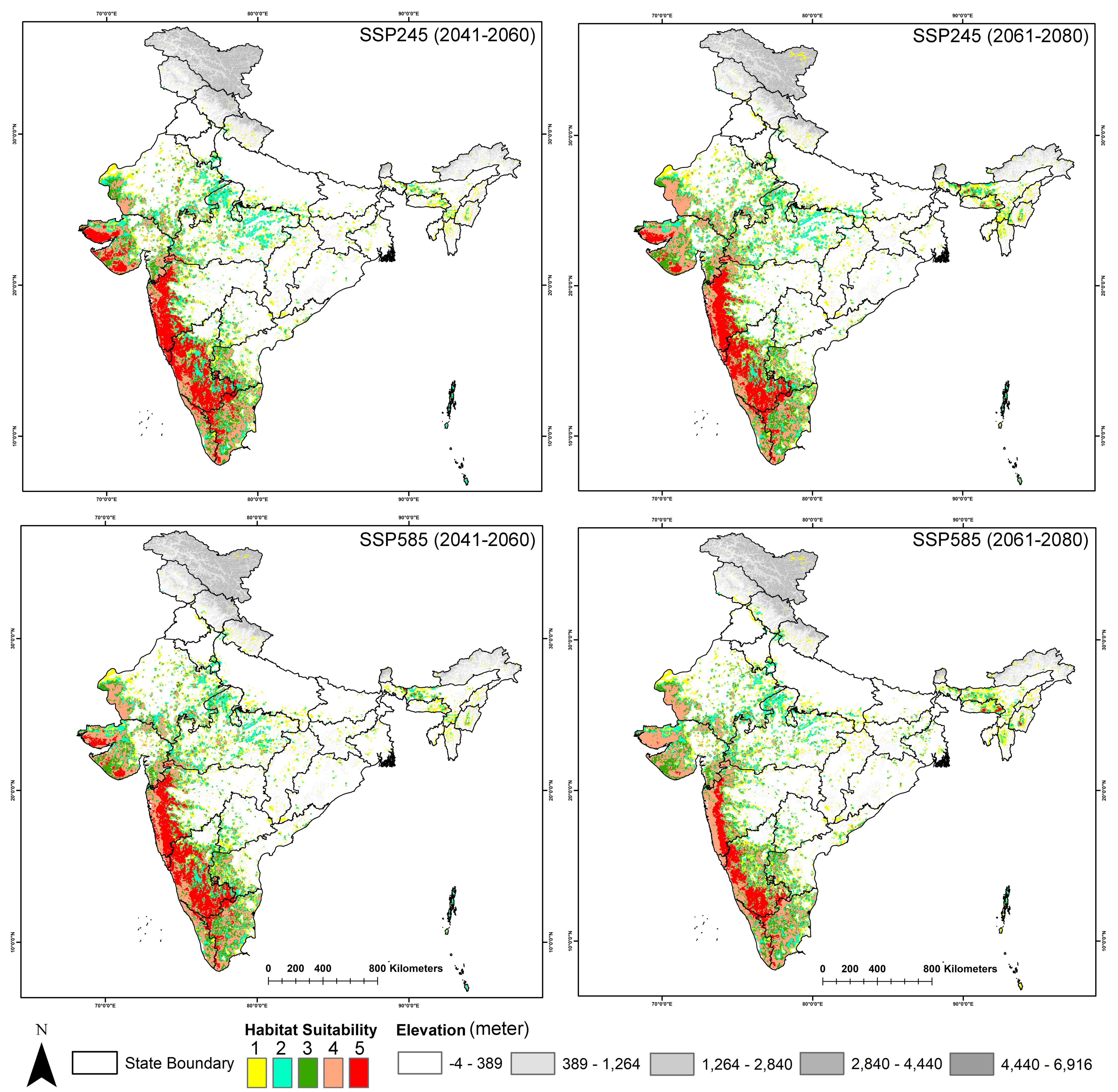

Supplement: S21 Fig — Here the ‘class 5’ determines the extremely suitable habitat extent. The administrative layer of the map was obtained from the DIVA-GIS website (https://diva-gis.org/data.html). The base layer of the maps is an elevation raster sourced from the SRTM website (http://srtm.csi.cgiar.org/srtmdata/) and was created using ArcGIS software. (TIF) [file pntd.0013464.s021.tif]

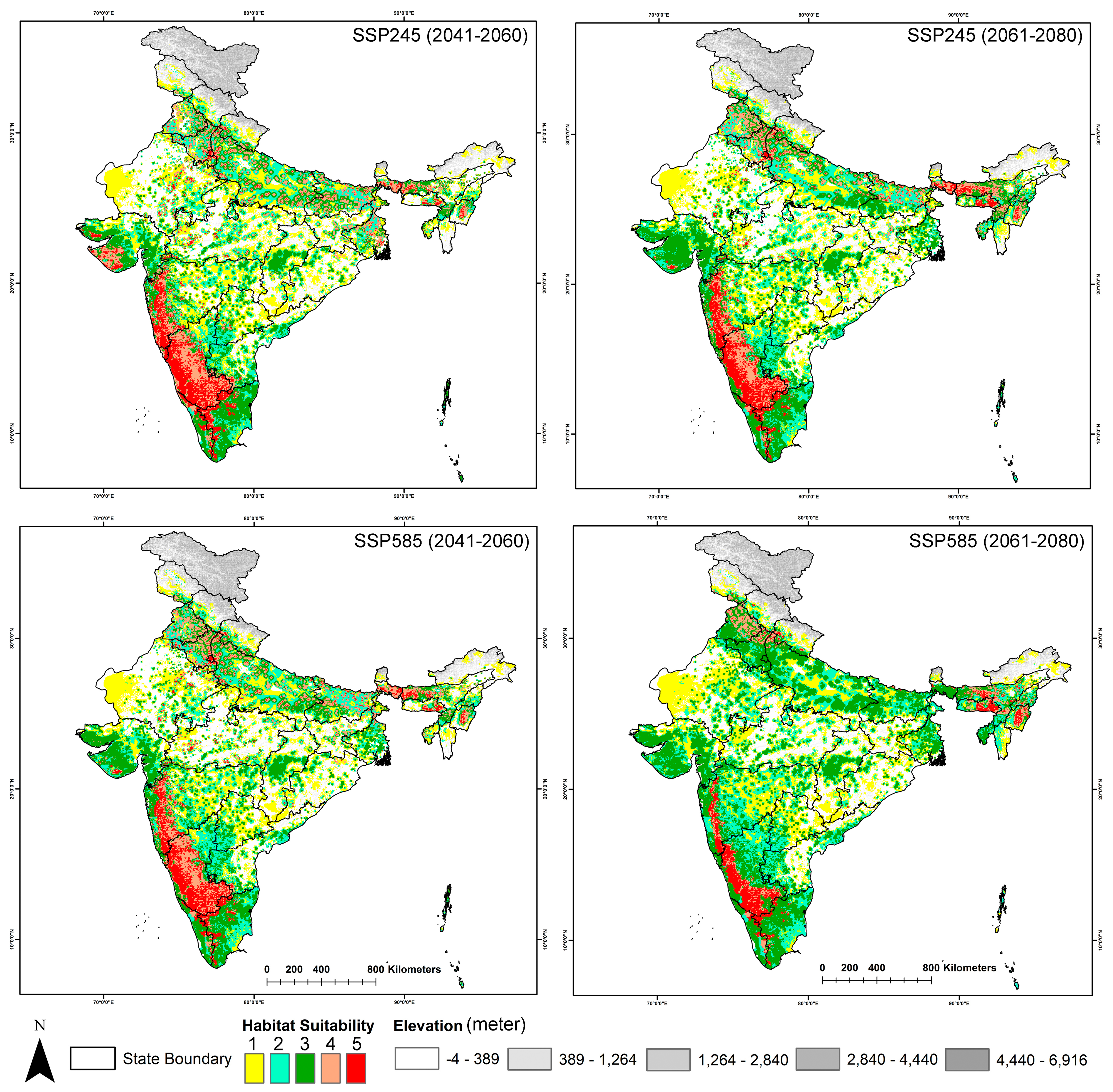

Supplement: S22 Fig — Here the ‘class 5’ determines the extremely suitable habitat extent. The administrative layer of the map was obtained from the DIVA-GIS website (https://diva-gis.org/data.html). The base layer of the maps is an elevation raster sourced from the SRTM website (http://srtm.csi.cgiar.org/srtmdata/) and was created using ArcGIS software. (TIF) [file pntd.0013464.s022.tif]

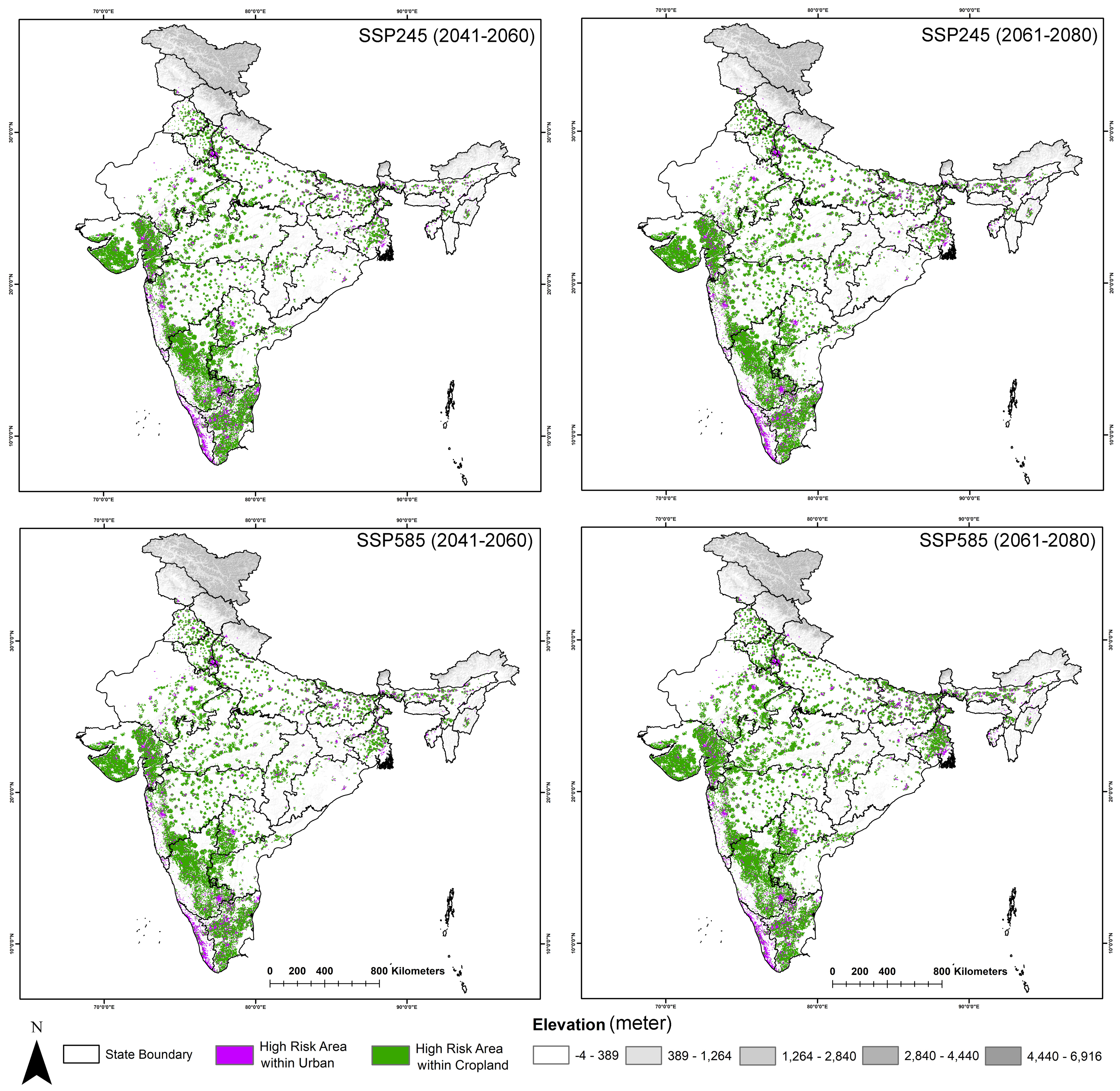

Supplement: S23 Fig — The administrative layer of the map was obtained from the DIVA-GIS website (https://diva-gis.org/data.html). The base layer of the maps is an elevation raster sourced from the SRTM website (http://srtm.csi.cgiar.org/srtmdata/) and was created using ArcGIS software. (TIF) [file pntd.0013464.s023.tif]

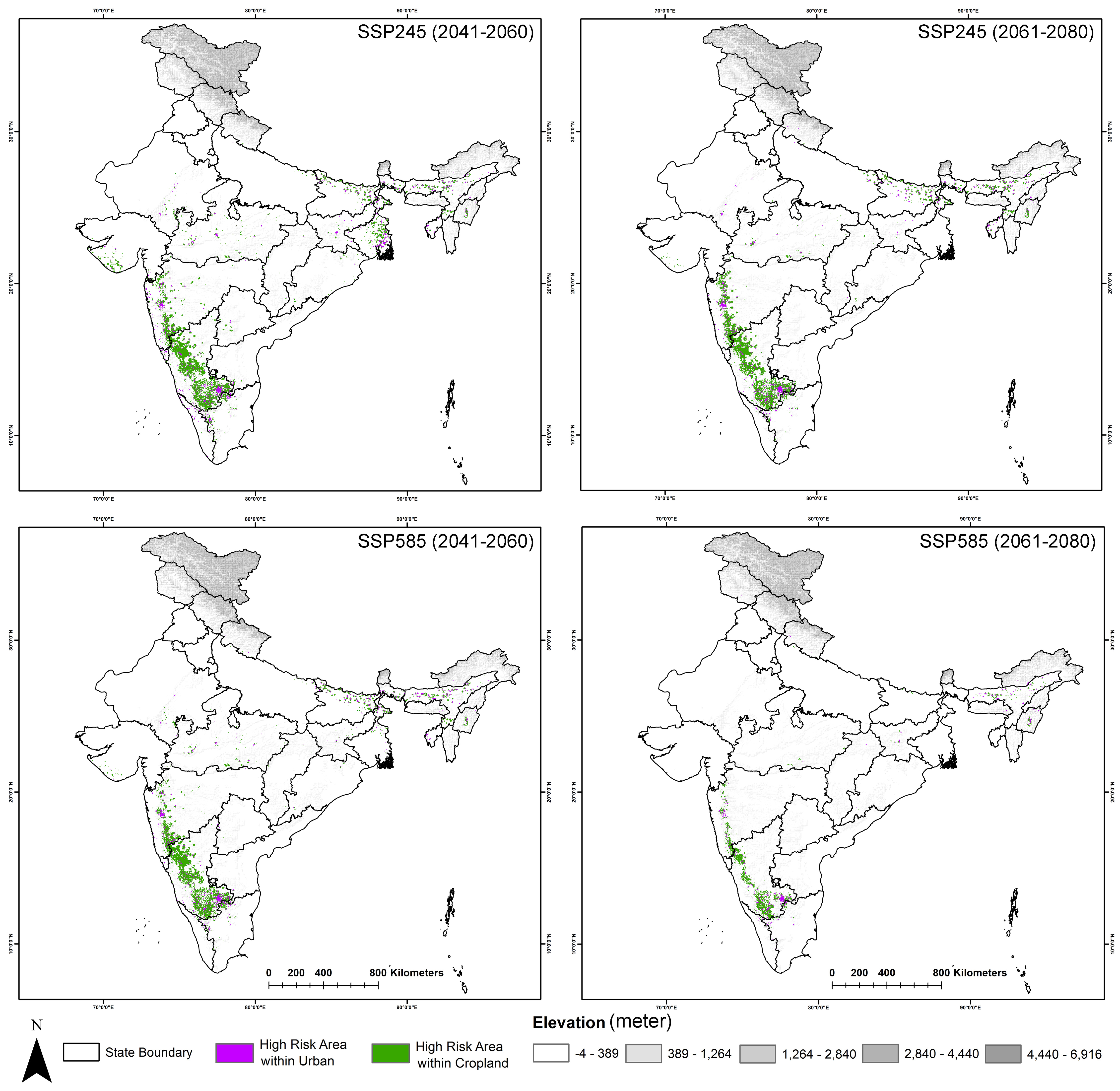

Supplement: S24 Fig — The administrative layer of the map was obtained from the DIVA-GIS website (https://diva-gis.org/data.html). The base layer of the maps is an elevation raster sourced from the SRTM website (http://srtm.csi.cgiar.org/srtmdata/) and was created using ArcGIS software. (TIF) [file pntd.0013464.s024.tif]

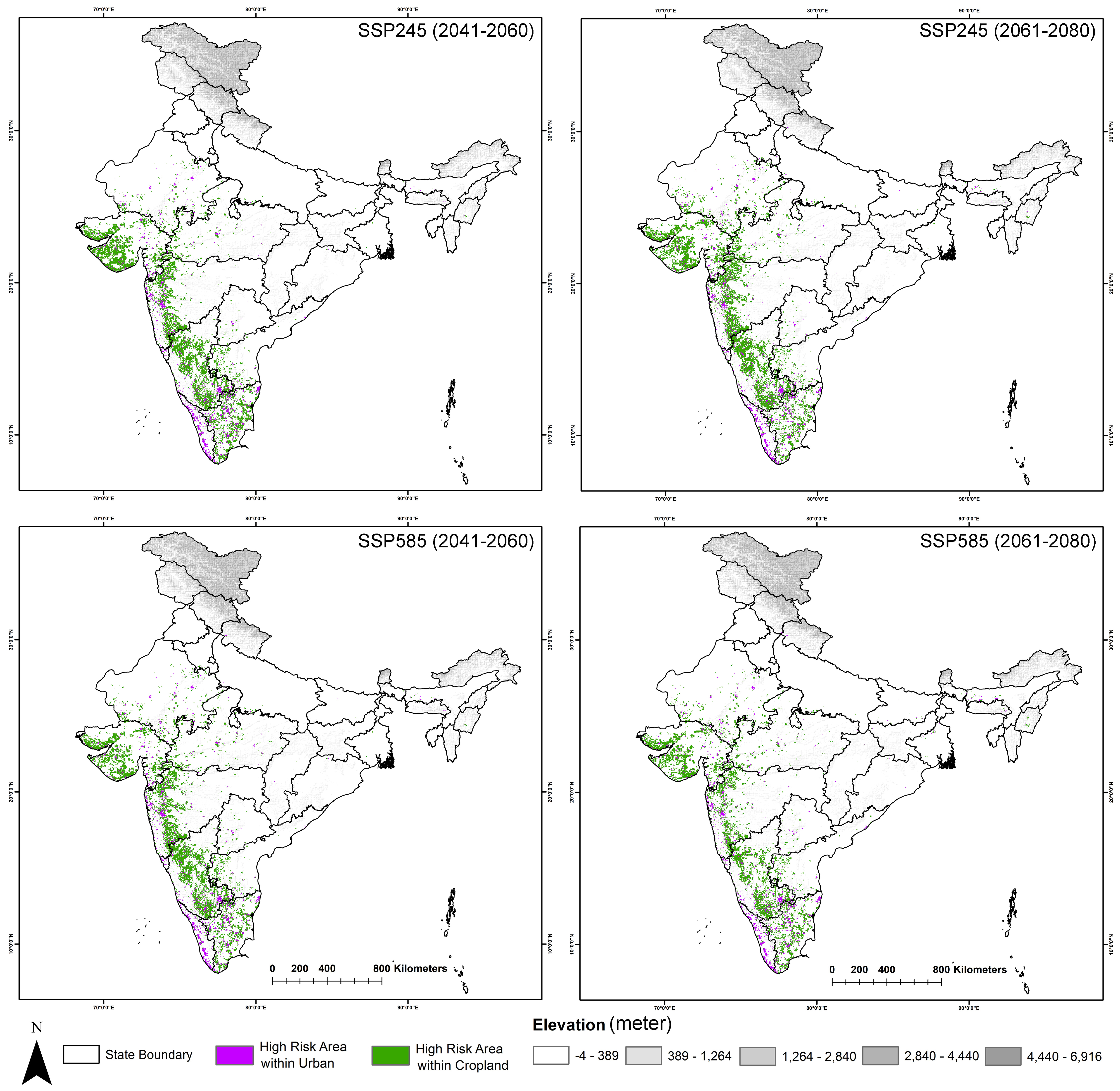

Supplement: S25 Fig — The administrative layer of the map was obtained from the DIVA-GIS website (https://diva-gis.org/data.html). The base layer of the maps is an elevation raster sourced from the SRTM website (http://srtm.csi.cgiar.org/srtmdata/) and was created using ArcGIS software. (TIF) [file pntd.0013464.s025.tif]

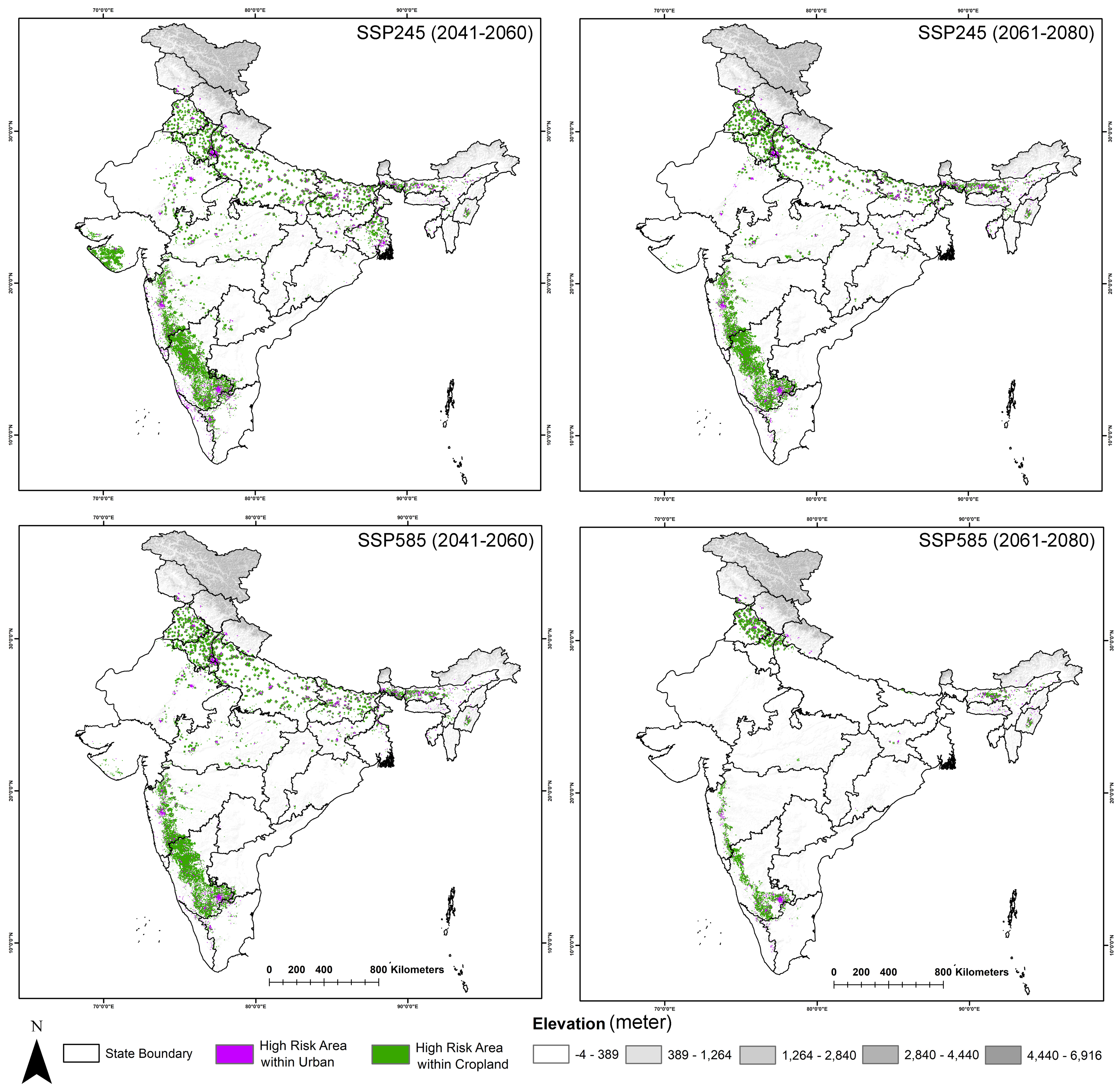

Supplement: S26 Fig — The administrative layer of the map was obtained from the DIVA-GIS website (https://diva-gis.org/data.html). The base layer of the maps is an elevation raster sourced from the SRTM website (http://srtm.csi.cgiar.org/srtmdata/) and was created using ArcGIS software. (TIF) [file pntd.0013464.s026.tif]

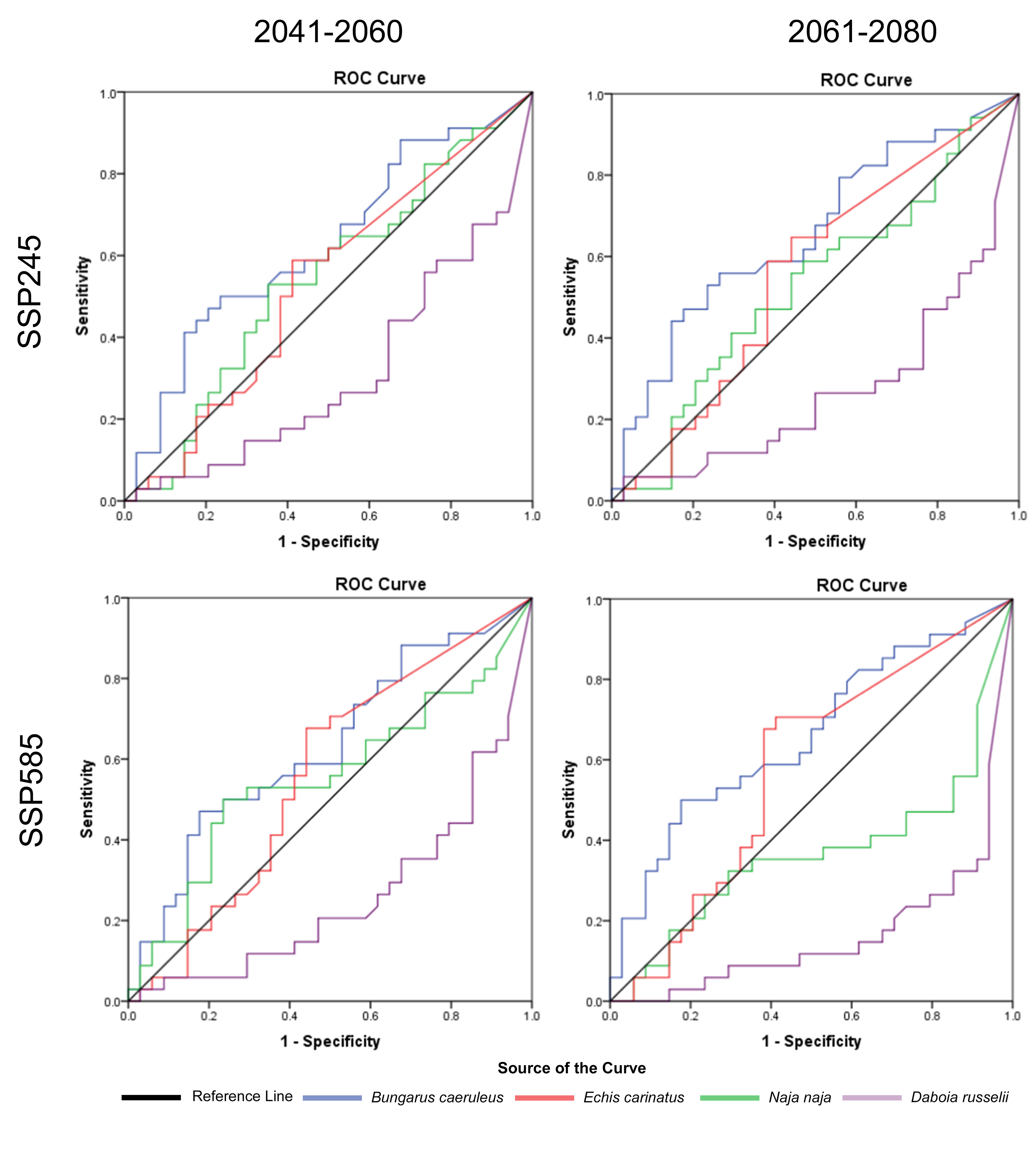

Supplement: S27 Fig — (TIF) [file pntd.0013464.s027.tif]

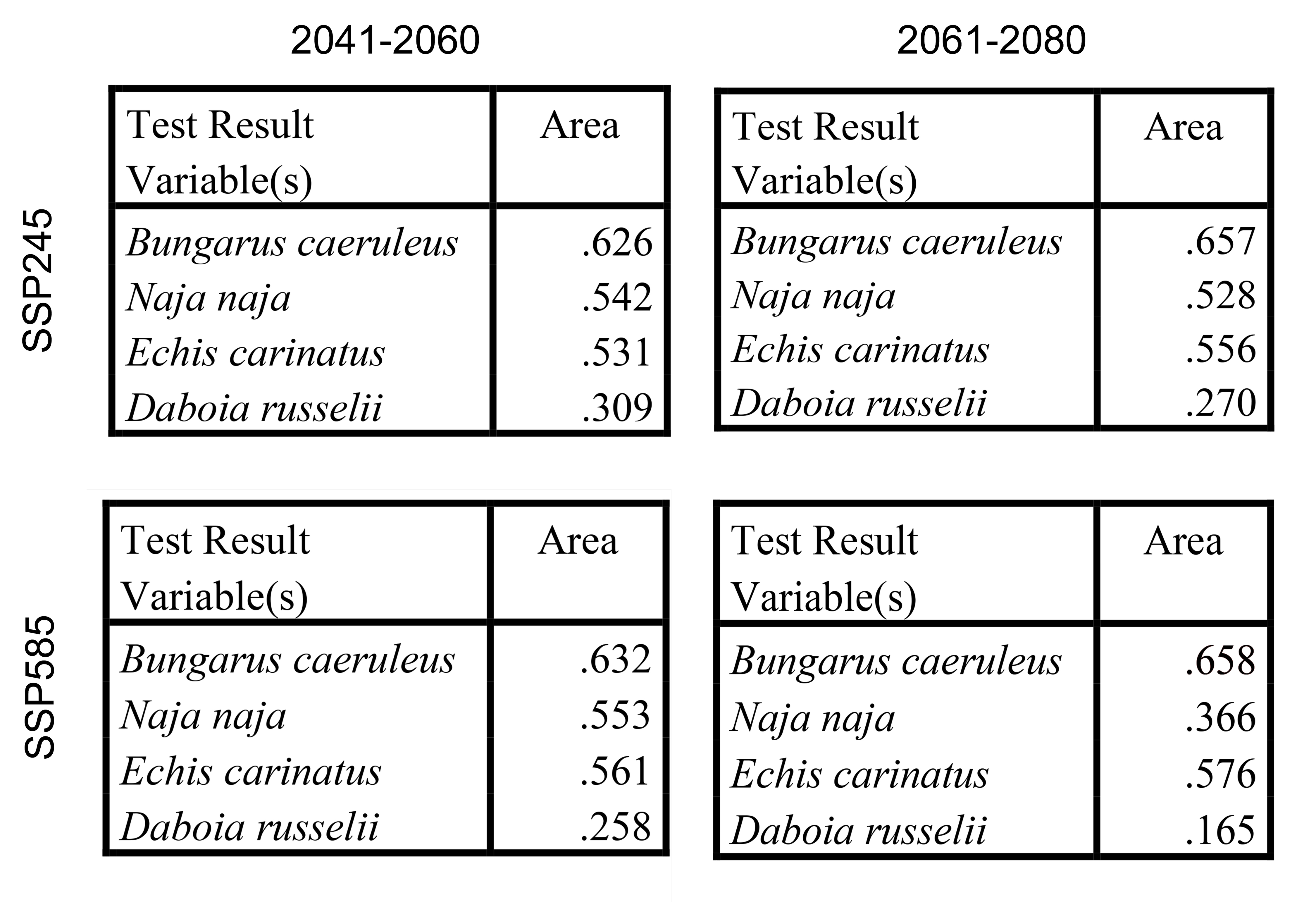

Supplement: S28 Fig — (TIF) [file pntd.0013464.s028.tif]

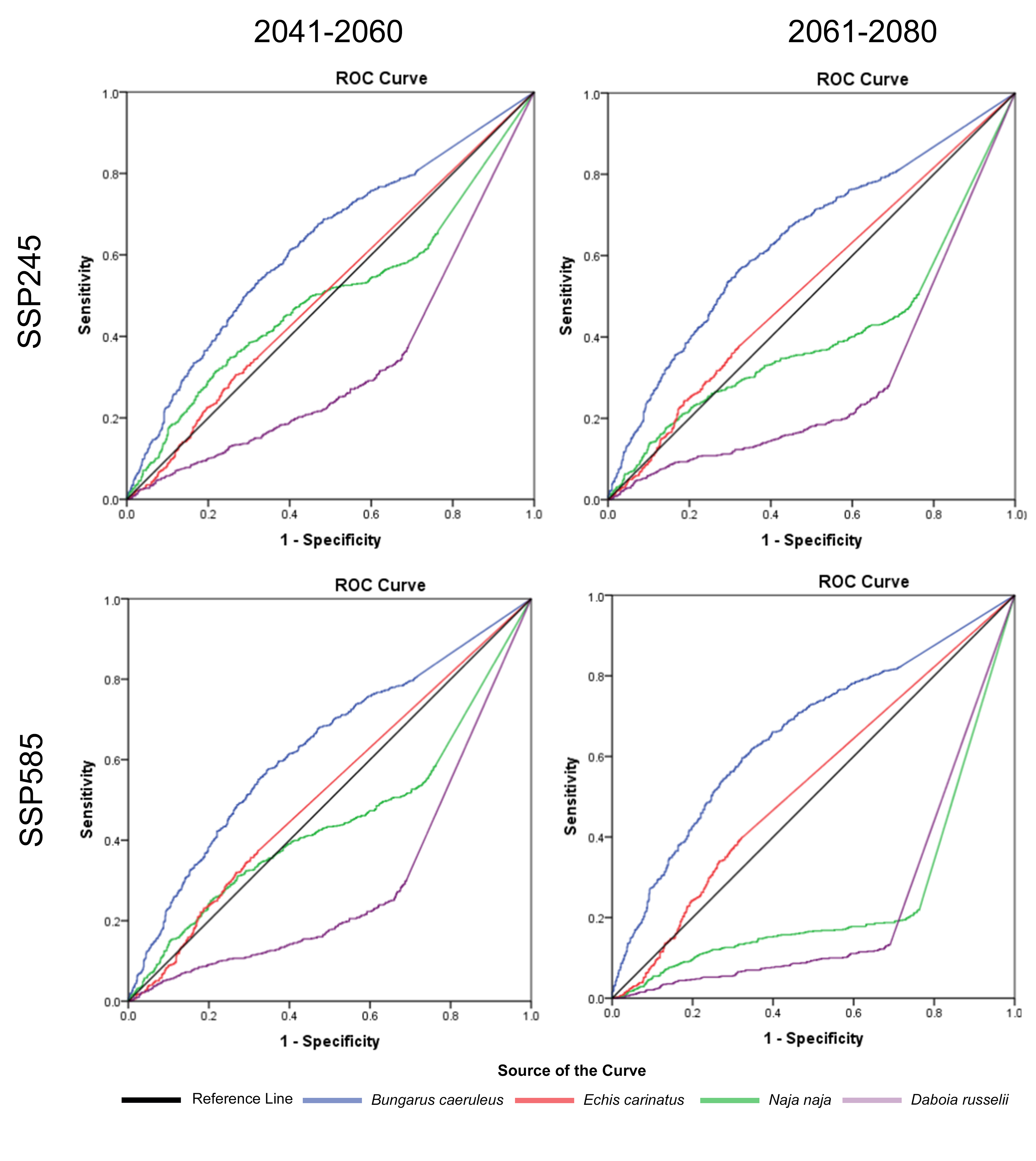

Supplement: S29 Fig — (TIF) [file pntd.0013464.s029.tif]

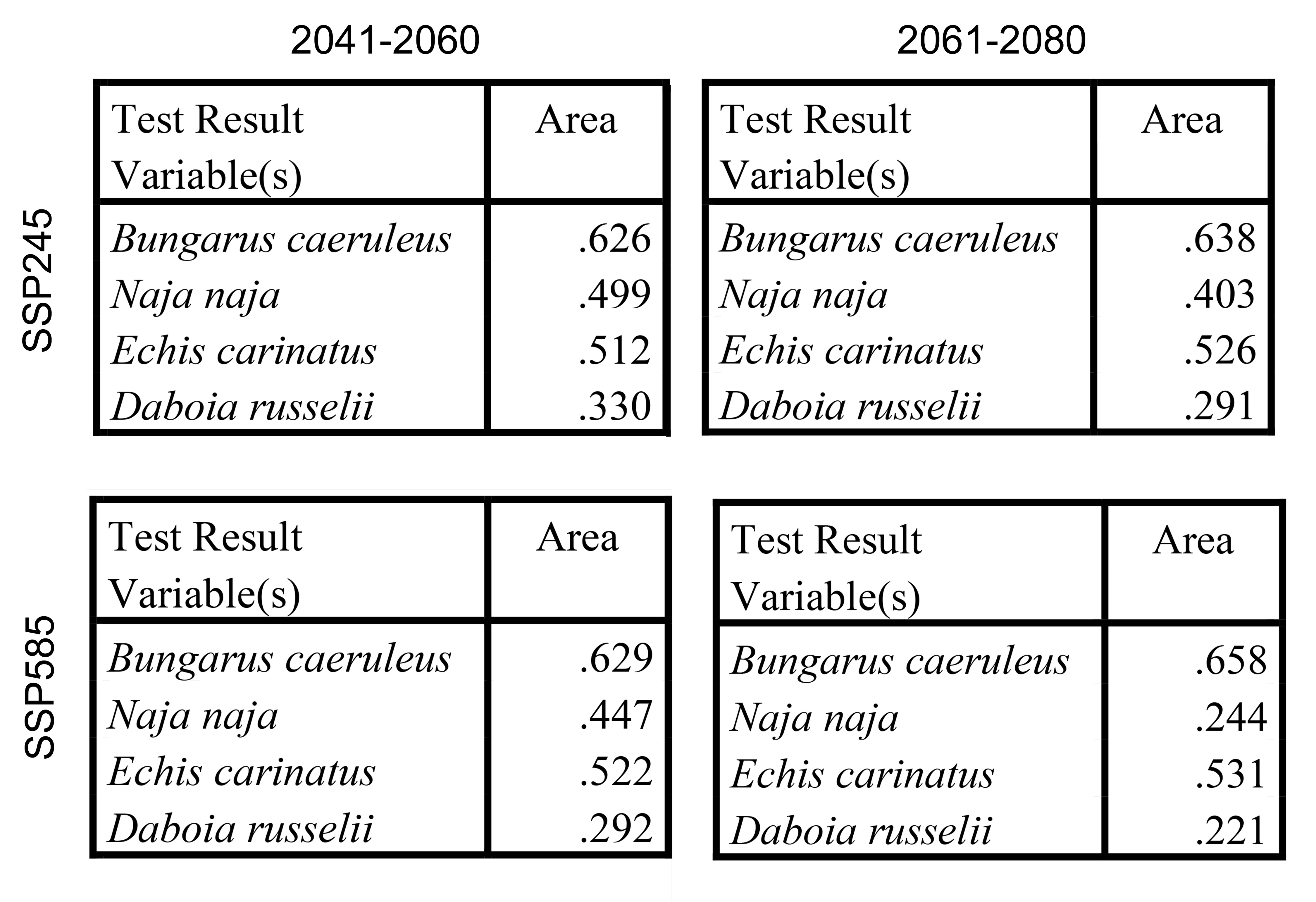

Supplement: S30 Fig — (TIF) [file pntd.0013464.s030.tif]
